# Early metabolic 18F-FDG PET/CT response of locally advanced squamous-cell carcinoma of head and neck to induction chemotherapy: A prospective pilot study

**DOI:** 10.1371/journal.pone.0200823

**Published:** 2018-08-16

**Authors:** Ulisses Ribaldo Nicolau, Victor Hugo Fonseca de Jesus, Eduardo Nóbrega Pereira Lima, Marclesson Santos Alves, Thiago Bueno de Oliveira, Louise De Brot Andrade, Virgilio Souza Silva, Paula Cacciatore Bes, Tadeu Ferreira de Paiva, Vinicius Fernando Calsavara, Andrea Paiva Gadelha Guimarães, Loureno Cezana, Paula Nicole Vieira Pinto Barbosa, Gislaine Cristina Lopes Machado Porto, Antônio Cássio Assis Pellizzon, Genival Barbosa de Carvalho, Luiz Paulo Kowalski

**Affiliations:** 1 Medical Oncology Department, A.C. Camargo Cancer Center, São Paulo, SP, Brazil; 2 Imaging Department, A.C. Camargo Cancer Center, São Paulo, SP, Brazil; 3 Pathology Department, A.C. Camargo Cancer Center, São Paulo, SP, Brazil; 4 Biostatistics Department, A.C. Camargo Cancer Center, São Paulo, SP, Brazil; 5 Radiotherapy Department, A.C. Camargo Cancer Center, São Paulo, SP, Brazil; 6 Head and Neck Surgery Department, A.C. Camargo Cancer Center, São Paulo, Brazil; UniversitatsSpital Zurich, SWITZERLAND

## Abstract

**Objective:**

The objective of this study was to assess the clinical value of 18-fluorodeoxyglucose positron emission tomography/computed tomography (^18^F-FDG PET/CT) after the first cycle of induction chemotherapy (IC) in locally advanced squamous cell carcinoma of the head and neck (LASCCHN).

**Methods and findings:**

A prospective, single-arm, single center study was performed, with patients enrolled between February 2010 and July 2013.Patients (n = 49) with stage III/IVA–B LASCCHN who underwent IC with taxanes, cisplatin, and fluorouracil were recruited. Staging procedures included loco-regional and chest imaging, endoscopic examination, and PET/CT scan. On day 14 of the first cycle, a second PET/CT scan was performed. Patients with no early increase in regional lymph node maximum ^18^F-FDG standard uptake value (SUV), detected using ^18^F-FDG PET/CT after first IC had better progression-free survival (hazard ratio (HR) = 0.18, 95%, confidence interval (CI) 0.056–0.585; p = 0.004) and overall survival (HR = 0.14, 95% CI 0.040–0.498; p = 0.002), and were considered responders. In this subgroup, patients who achieved a reduction of ≥ 45% maximum primary tumor SUV experienced improved progression-free (HR = 0.23, 95% CI 0.062–0.854; p = 0.028) and overall (HR = 0.11, 95% CI 0.013–0.96; p = 0.046) survival.

**Conclusions:**

These results suggest a potential role for early response evaluation with PET/CT examination in patients with LASCCHN undergoing IC. Increased regional lymph node maximum SUV and insufficient decrease in primary tumor uptake predict poorer outcomes.

## Introduction

Phase III trials and meta-analyses published during the 1990s established radiotherapy combined with chemotherapy (generally administered concurrently) as standard treatment for patients demanding organ-preservation or harboring unresectable locally advanced squamous-cell carcinoma of the head and neck (LASCCHN) [1–8].

In the last decade, randomized trials comparing induction chemotherapy (IC) regimens where taxanes were added to cisplatin and fluorouracil (TPF) to the previous standard, cisplatin plus fluorouracil (PF), regimen demonstrated improved outcomes favoring taxane-containing triplet regimens and led to the introduction of IC with triplet regimens followed by radiotherapy as a treatment option for patients with LASCCHN. However, despite their greater efficacy, grade 3 or higher toxicity, occurring in more than 70% of patients, has limited the use of triplet regimens [9–12].

Clinical, endoscopic, and radiological tumor response evaluation to IC-CT RT for LASCCHN is usually performed after 2-IC cycles, based on the anatomic World Health Organization (WHO) or modified WHO (mWHO) criteria, in which a partial response requires a bi-dimensional decrease in the target lesion of 50% and 25%, respectively, and neither increase in the regional neck lymph node lesions, nor evidence of distant metastasis. Patients whose primary tumors or regional lymph nodes experience an enlargement higher than 25% in their bi-dimensional diameters during IC experience disease progression according to the WHO criteria and they are usually treated with salvage surgery. Accordingly, patients developing distant metastasis are considered as patients harboring non-responsive tumors and are often treated with palliative systemic therapy. Patients with primary tumors that exhibit at least a partial response, according to computed tomography (CT) or magnetic resonance imaging (MRI) evaluation, are usually treated using sequential radiotherapy with concurrent chemotherapy or biotherapy (CBRT) [9–10,13–15].

Concerns regarding the toxicity profile of triple IC regimens suggest that early response evaluation could identify patients harboring non-responsive tumors, who could be spared from toxicity by prompt initiation of an alternative treatment [16–17]. Moreover, the identification of a subgroup of patients with HPV-related oropharyngeal squamous-cell carcinoma (OPSCC) with favorable prognosis raised the prospect of safe de-intensification protocols for some patients, according to early tumor response evaluation [18–23].

Positron emission tomography/Computed tomography (PET/CT) using the glucose analog fluorodeoxyglucose F-18 (^18^F-FDG) is employed in head and neck squamous-cell carcinoma (HNSCC) for staging, management of unknown primary tumor, post-radiotherapy response evaluation, and patient surveillance [24–32]. ^18^F-FDG PET/CT has demonstrated ability to identify groups of patients with other malignant diseases with better clinical outcomes early in the course of treatment [33–36]. Therefore, we conducted a prospective trial to test the hypothesis that an early decrease in ^18^F-FDG uptake, measured by standard uptake value (SUV) change, detected using ^18^F-FDG PET/CT after the first cycle of IC could identify patients with improved clinical outcomes. The aim of the trial was to examine the relationship between changes in primary tumor maximal SUV from baseline to post-cycle 1 ^18^F-FDG PET/CT with progression-free survival (PFS).

Furthermore, we tested whether early functional ^18^F-FDG PET/CT imaging could replace the standard post-second-cycle anatomical WHO and mWHO evaluation for patients treated for LASCCHN using IC followed by CBRT.

## Materials and methods

### Study design and compliance with ethical standards

The study was a prospective, single-arm, single center trial; it was reviewed and approved by the Antonio Prudente Foundation /AC Camargo Cancer Center Research Board on November 10, 2009 under registration number 1288/09. An every 6 months report for AC Camargo Cancer Center Research Board was performed during enrollment patients period. The trial was designed and approved in 2009, and followed all the regulatory Brazilian law demands for prospective trial registering requirements involving human subject research at that period. The trial is registered at the Brazilian Clinical Trials Registry (ReBEC) RBR-9WWSTD. The trial was registered in the ReBEC after opening the period for patients enrollment because the Brazilian system for clinical trials Registry started its activities on 2010. Written informed consent was obtained from all patients. All patients who signed the informed consent for the trial participation are analyzed in the present report. The authors confirm that all on going and related trials for this intervention are registered. All procedures performed in this study were in accordance with the ethical standards of the institutional research committee, and with the 1964 Helsinki declaration and its later amendments.

### Population

Between February 2010 and July 2013 eligible patients were enrolled, with a median follow- period of 44.3 months at the time of this analysis. Patient eligibility criteria for this study included: a histologically confirmed diagnosis of untreated stage III or IVA/B HNSCC, according to the AJCC staging system (6th edition); tumors arising from the oropharynx, hypopharynx, larynx, or oral cavity; a minimum age of 18 years; performance status, ECOG 0–1; and absence of impeditive major co-morbidities or organ dysfunctions.

### Imaging protocols

All the imaging exams were performed in the same technical equipment.

CT scans were performed using a 16-section multidetector row CT scanner (Philips Brilliance Big Bore, Philips Healthcare, Cleveland, OH). Head and neck CT was performed after administration of 80–120 mL of a nonionic contrast (Optiray 320; Mallinckrodt). MRI examinations were performed at 1.5 T MR scan (SignaHDxT system, GE Medical Systems, Milwaukee, WI). MRI protocol included axial T1-and T2-weighted images, coronal T2-weighted fat saturated images and T1-weighted images after intravenous administration of gadolinium-based paramagnetic contrast agent (10 ml of gadoversetamide, Optimark® Mallinckrodt). Two experienced medical radiologists reviewed all CT and MRI images.

Whole body ^18^F-FDG PET/CT imaging was performed 60 minutes after intravenous injection of 0.154 mCi/Kg of ^18^F-FDG (IPEN-CNEN). Patients with normal blood glucose levels were injected at rest and after at least 4 hours of fasting. Both low dose CT and dedicated PET imaging protocols, starting from the head to the proximal thigh, were acquired in a 64 channel PET/CT (Gemini TOF, Philips Medical Systems). Nuclear medicine specialists with 15 and 25 years of experience reviewed images and SUV data in concordance.

## Tumor diagnosis, staging, treatment plan, and response evaluation

Patients with stage III–IVB LASCCHN were prospectively evaluated and underwent IC with TPF followed by CBRT. Staging procedures included physical and endoscopic examination, and CT or MRI of primary tumor, neck, and chest. A baseline PET/CT examination was performed for all patients, and the pre-treatment maximal SUV of primary tumors and regional neck lymph node metastases were recorded. It is noteworthy to point that in the mean time of development of this study a policy of routine HPV testing in primary OPSCC was implemented in our center. In this way, patients with OPSCC with available tissue samples were tested for HPV by p16 staining or high-risk HPV genotyping [23].

### Induction chemotherapy and radiosensitizing therapy

Patients were treated with 3–4 cycles of IC with a taxane (paclitaxel, 175mg/m^2^or docetaxel, 75mg/m^2^) combined with 75–100 mg/m^2^ of cisplatin on day 1, and continuous infusion of 750 mg/m^2^5-fluorouracil daily for 5 days. Radiosensitizers included weekly carboplatin (area under the curve, 1.5), and 100 mg/m^2^cisplatin every 3 weeks.

### Radiotherapy

Intensity modulated radiotherapy (IMRT) or 3-dimensional conformal therapies were allowed for study entry. Treatments were performed in linear accelerators with 6 MV photons. Maximum doses for gross tumor volume (macroscopic disease) were 66–70 Gy, over 6–8 weeks.

### Tumor response evaluation, experimental procedures and patients follow-up

All cases were assessed according to standard WHO or mWHO criteria for disease evaluation. After the second cycle, tumor response was assessed by the treating physician, using physical and endoscopic examination, and primary tumor/neck CT or MRI. Patients with at least partial tumor shrinkage, according to WHO or mWHO criteria, were selected for a further cycle of IC followed by CBRT.

### Experimental imaging procedures

On the fourteenth day of the first IC cycle (D14-C1-IC), a second PET/CT was performed and the maximum SUVs in the primary tumor and regional neck lymph node metastasis recorded. Treating physicians and patients were blinded to these findings.

### Patients follow-up

After treatment, patients were initially evaluated by physical examination and laboratory monitoring for toxicity and tumor response, and then monthly. CT or MRI was performed within 2 months after the end of radiotherapy. Follow-up visits for efficacy and safety assessments were scheduled every 1–2 months during the first year, every 2–3 months during the second and third years, and every 6 months thereafter. After treatment completion, head and neck, and chest imaging examinations were performed every 4 months in the first year, every 4–6 months in the second year, and yearly afterwards. Efforts were made to confirm pathological tumor-recurrence and progressive disease in cases if clinically demanded, according to current institutional clinical practice.

### Statistical analyses

Patient characteristics are expressed as absolute and relative frequencies for qualitative variables, and as median and range for quantitative variables. The primary outcome of the study was progression-free survival. For patients with any reduction of neck lymph nodes’maximum SUV and no distant disease progression, we compared PFS of two group of patients according to the extent of primary tumor maximal SUV reduction following the first cycle of IC. The two groups were generated using a post-hoc derived cutoff of primary tumor maximal SUV reduction according to maximum standardized log-rank statistics [37]. The maximally selected log-rank statistics for cut-points between 5% and 95% percentiles of continuous variables (variation in primary tumor maximum SUV) were considered. As secondary outcome, we assessed the difference in overall survival according to primary tumor maximum SUV reduction following the first cycle of IC. We also evaluated PFS and OS according to WHO and mWHO response criteria. Moreover, a sub-group analysis was performed in OPSCC patients. The Kaplan-Meier estimator was used for survival analysis, and log-rank tests were applied to compare survival distributions between groups. The Cox proportional hazards model was used to describe the relationship between time-point variables and covariates [38]. Proportionality assumption was evaluated using the Schoenfeld residuals test [39–40]. In all cases, there was evidence that covariates had a constant effect over time. Considering the relatively small sample size, the primary tumor maximum SUV reduction cut-off, derived from the maximum of the standardized log-rank statistic, was subjected to non-parametric 10,000 bootstrap sampling with replacement for internal validation, applied to PFS and OS. All statistical analysis were performed using either the R statistical package or SPSS v21.0 software.

### Protocol deviations

One patient has performed the baseline PET/CT in a different equipment and was originally evaluated by an external nuclear medicine specialist due to an initial diagnosis investigation initiated in other hospital, and it was not performed in the pretreatment period in our hospital as demanded by protocol. This PET/CT imaging was available for evaluation of our nuclear medicine specialist evaluation at the time of the performance the post-cycle 1 IC experimental PET/CT. Two patients did not perform the D14 cycle 1 IC PET/CT and were not be included in this analysis for the reasons described in the results section. One patient performed the planned D14 cycle 1 IC PET/CT at the day 25 cycle 1 IC due to a upper aerodigestive tract infection that led to postpone the second IC cycle. The experimental post-cycle 1 PET/CT of this patient was performed before the cycle 2, after clinical recovery from infection. Seven patients underwent alternative post-TPF IC concurrent radiosensitizing therapy in accordance with assistant physician choice, which included weekly (instead of every 3 week) cisplatin for 3 patients, weekly cetuximab (at usual cetuximab loading dose 400mg/m2 followed by 250mg/m2 weekly infusion) for 3 patients, and exclusive radiotherapy for 1 patient.

## Results

Forty-nine consecutive patients diagnosed with LASCCHN were enrolled in this study. Table 1 shows the demographic and clinical characteristics of the patient population. The majority of patients were male, and smokers or former smokers. The most frequent primary tumor site was oropharynx, and tumors were often HPV-related. HPV status was determined in 26 (63%) of the 41 OPSCC patients. Forty patients presented with stage IV disease (Table 1 and S1 Table).

**Table 1:** Demographic and clinical characteristics of the study population.

Characteristic	Patients (49) N (*%*)
**Sex**	
**Male**	44 (90)
**Female**	5 (10)
**Age (years)**	
**Median (range)**	55 (39–74)
**<50**	13 (27)
**50–59**	24 (49)
**60–69**	10 (20)
**≥70**	2 (4)
**Smoking history**	
**Never**	14 (29)
**Former**	15 (31)
**Current**	20 (41)
**Tumor site**	
**Oropharynx**	41 (84)
**Larynx**	4 (8)
**Hypopharynx**	4 (8)
**Staging**	
**III**	9 (18)
**IVA**	30 (61)
**IVB**	10 (20)
**Tumor staging**	
**T1–2**	16 (33)
**T3**	22 (45)
**T4**	10 (20)
**Tx**	1 (2)
**Lymph Node staging**	
**N0–1**	11 (22)
**N2**	31 (63)
**N3**	7 (14)
**HPV status (oropharynx only)**	
**Positive**	19 (46)
**Negative**	7 (17)
**Unknown**	15 (37)

Demographic and clinicopathological characteristics of the entire patient population (N = 49).

Staging followed AJCC recommendations (6^th^ edition).

Most patients received three cycles of IC, and docetaxel was the most frequently employed taxane. IMRT and concurrent carboplatin were the most frequently administered treatments (S2 Table).

### Assessments and outcomes

Two patients were not included in the primary outcome analysis because they were unable to undergo early(D14-C1-IC) ^18^F-FDG PET/CT examination because of post-cycle 1 grade 4 hyponatremia (1 patient), and synchronous colorectal cancer diagnosed by the baseline PET/CT, demanding an immediate colonic resection, with subsequent IC followed by CBRT for LASCCHN treatment (1 patient). Both patients were alive and disease free at the time of this analysis.

**Fig 1:**
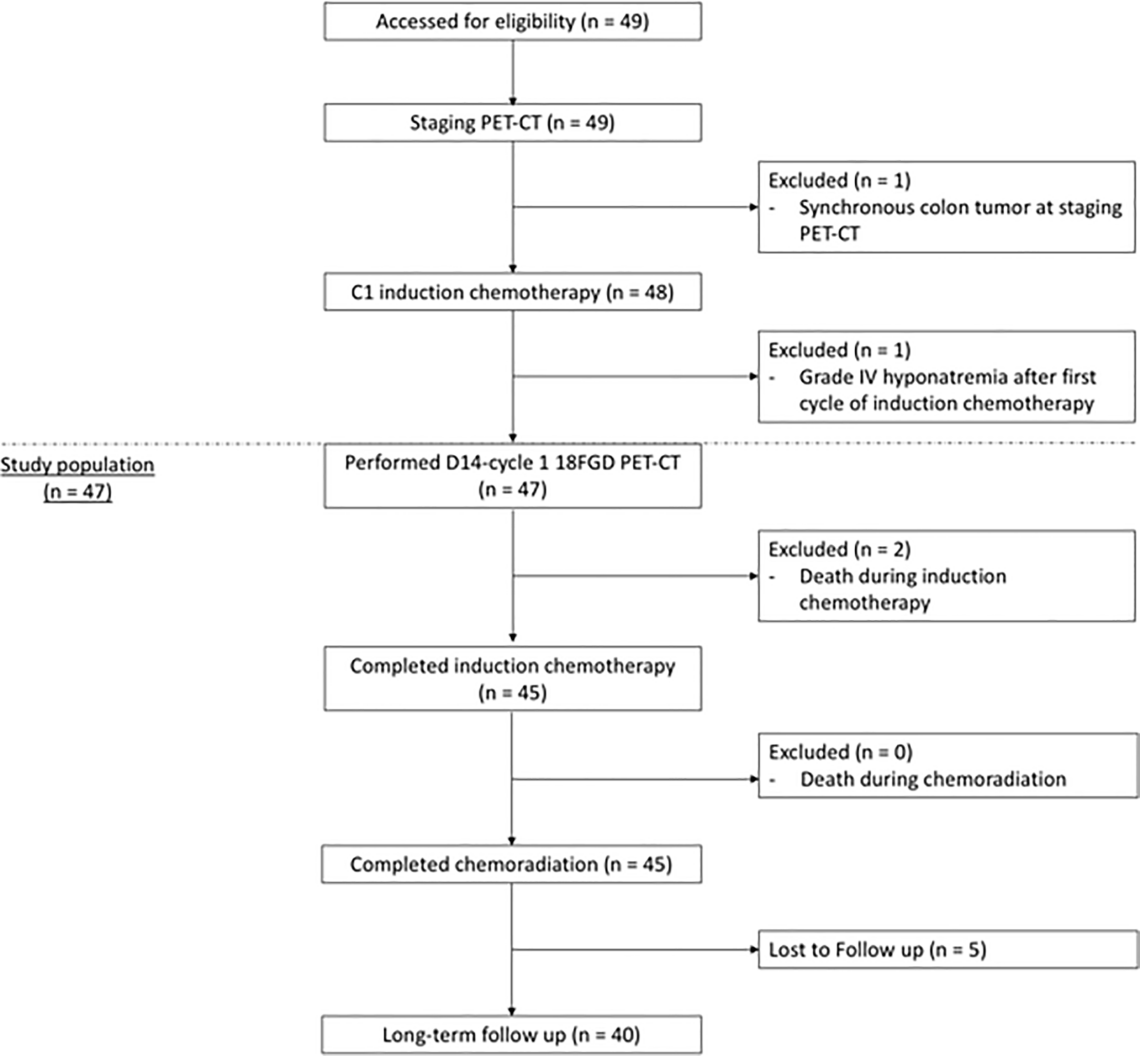
The Consort flowchart descriptions of the entire study populations.

**Fig 2:**
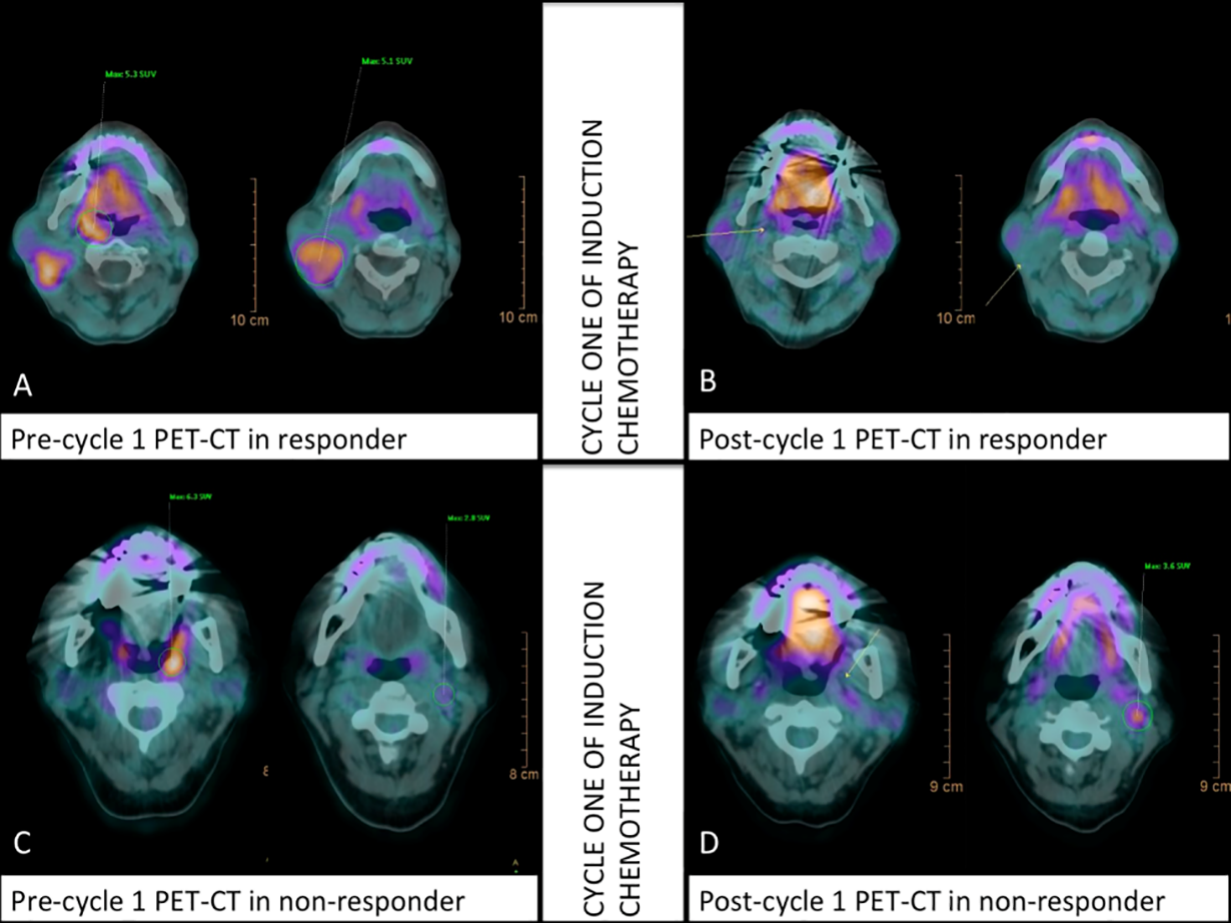
Two patients presenting metabolic responsive and non-responsive tumors/ neck lymph node after once IC cycle. Pretreatment PET/CT(A) and post-treatment PET/CT(B) in patient harboring oropharyngeal tumor with complete metabolic response at 14^th^ day, cycle 1 of induction chemotherapy (SUV variation from 5.1 to 0.0 and 5.3 to 0.0 in lymph node and primary tumor respectively). Pretreatment PET/CT(C) and post-treatment PET/CT(D) in patient harboring non-responsive oropharyngeal tumor with Increased metabolic response at 14^th^ day, cycle 1 of induction chemotherapy (neck lymph node SUV variation from 2.8 to 3.6, despite achieving a complete metabolic response in primary tumor).

Among the remaining 47 patients, 83% and 92% had morphologically responsive tumors, according to standard WHO and mWHO criteria, respectively. Patients whose tumors achieved at least partial response according to mWHO criteria received a third cycle of IC followed by CBRT. On D14-C1-IC PET/CT, nine patients showed SUV elevation in lymph nodes (six patients), primary tumors (two patients), or both primary tumor and lymph nodes (one patient). Hence, 40 (85%) patients demonstrated a decrease in lymph node SUV, and were considered to have metabolically responsive tumors. Table 2 illustrates individual patient responses according to PET/CT (change in primary tumor and regional lymph node maximum SUV), WHO, and mWHO criteria during IC. The Fig 1 describes the entire population consort flowchart. In Fig 2, we report two patients harboring responsive and non-responsive tumors according to D14 cycle 1 IC PET/CT evaluation, respectively.

**Table 2:** Response to induction chemotherapy according to PET/CT.

Patient no.	Site	Clinical Staging	^18^F-FDG (max SUV)–T			^18^F-FDG (max SUV)—N			Response (WHO)	Response (mWHO)
			Baseline	Day 14	Change (%)	Baseline	Day 14	Change (%)		
**1**	oropharynx	IVA	7.21	6.34	-13	4.3	3.4	-21	yes	yes
**2**	oropharynx	IVA	16.07	6.79	-58	7.6	4.88	-36	no	no
**3**	oropharynx	IVA	3.32	2.83	-15	7.3	4.08	-44	yes	yes
**4**	oropharynx	IVA	7	4.3	-39	4.55	2.22	-51	no	no
**5**	hypopharynx	IVB	13.7	6.43	-53	5.56	5.81	4	no	yes
**6**	oropharynx	IVA	12.9	10.4	-19	6.5	3.47	-47	yes	yes
**7**	Larynx	III	9.97	4.76	-53	0	0	0	yes	yes
**8**	oropharynx	III	6.52	4.91	-25	0	0	0	yes	yes
**9**	oropharynx	IVB	6.95	5.07	-27	4.14	3.47	-16	yes	yes
**10**	oropharynx	IVA	9.19	5	-46	9.29	5.8	-38	yes	yes
**11**	oropharynx	III	12.27	4.9	-60	6.11	3.1	-49	yes	yes
**12**	oropharynx	IVA	14	5.9	-58	7.5	0	-100	yes	yes
**13**	hypopharynx	IVA	10.1	11	9	8.4	8.3	-1	yes	yes
**14**	hypopharynx	IVA	12.8	5.5	-57	5.2	3.6	-31	yes	yes
**15**	Larynx	IVA	6.1	3.7	-39	10	4.4	-63	yes	yes
**16**	oropharynx	IVA	15.2	4.6	-70	5.9	3.7	-37	yes	yes
**17**	oropharynx	IVA	8.9	9.9	11	4.9	9.6	96	yes	yes
**18**	oropharynx	IVA	8.8	1.9	-79	7.2	1.7	-77	yes	yes
**19**	oropharynx	III	8.3	4.4	-47	0	0	0	yes	yes
**20**	Larynx	IVA	7.9	0	-100	17.1	6.6	-61	no	no
**21**	oropharynx	IVA	9.8	8.9	-9	6.9	4.6	-33	yes	yes
**22**	oropharynx	IVA	6.9	6.9	0	9	6.8	-24	no	yes
**23**	oropharynx	IVA	5.9	3.8	-36	4.3	0	-100	yes	yes
**24**	oropharynx	IVA	9.6	4.6	-52	8	5.3	-34	yes	yes
**25**	oropharynx	IVA	7	5.2	-36	5.6	5.3	-5	no	yes
**26**	oropharynx	IVA	2.8	0	-100	3.3	4.5	36	yes	yes
**27**	oropharynx	III	7.7	4.1	-47	6.7	0	-100	yes	yes
**28**	oropharynx	IVB	17.2	11.5	-33	4.6	5.7	24	yes	yes
**29**	oropharynx	IVB	8.2	0	-100	0	0	0	yes	yes
**30**	oropharynx	IVA	6.9	0	-100	4.3	4.2	-2	yes	yes
**31**	oropharynx	III	3.7	2.8	-24	8.5	3	-65	yes	yes
**32**	oropharynx	III	7.3	4.4	-40	6.2	2.7	-56	yes	yes
**33**	oropharynx	IVA	14	4.5	-68	14.4	5.9	-59	yes	yes
**34**	oropharynx	IVB	5.8	3.9	-33	2.2	2.9	32	no	no
**35**	oropharynx	IVB	8.2	0	-100	13.9	8.5	-38.8	yes	yes
**36**	oropharynx	IVB	9.3	5	-46.2	9.7	5	-48.45	yes	yes
**37**	oropharynx	III	6.3	0	-100	2.8	3.6	28.57	yes	yes
**38**	oropharynx	IVA	6.1	0	-100	6.3	2.4	-60.65	yes	yes
**39**	oropharynx	IVA	13.9	7.7	-44.6	9.5	5	-47.36	yes	yes
**40**	oropharynx	IVB	5.3	0	-100	5.4	0	-100	yes	yes
**41**	hypopharynx	III	13.1	3.2	-75.5	2	1.6	-20	yes	yes
**42**	Larynx	IVB	5.3	3.6	-32	9.8	7.5	-23.4	yes	yes
**43**	oropharynx	IVA	9.3	6.3	-32.2	3.3	6.3	96.8	no	no
**44**	oropharynx	IVA	4.4	0	-100	6.7	0	-100	yes	yes
**45**	oropharynx	IVB	4.7	5.2	10.6	3.7	2.9	-21.6	yes	yes
**46**	oropharynx	IVA	4.7	3.2	-31.9	6.5	4.3	-33.8	yes	yes
**47**	oropharynx	IVA	5.8	3.2	-44.8	3.4	2.3	-32.3	yes	yes

Change in primary tumor and maximum standard uptake value (SUV) of regional lymph nodes after the first induction chemotherapy (IC), and WHO and mWHO criteria for each individual patient (after the second cycle of IC). N = 47.

After a median follow-up of 44.3 months (range, 2.0–70.6 months), 16 patients experienced progressive disease. There were 12 loco-regional, three distant, and one simultaneous loco-regional and distant progression of disease. Median PFS was not reached, and 3-year PFS was 69%. Ten patients died; all deaths were cancer-related. Median OS was not reached, and 3-year OS was 75%. Median follow-up was 41.6 months for surviving patients (range, 9.5–70.6 months).

Patients with responsive tumors experienced higher PFS than patients with non-responsive tumors according to morphological WHO criteria (log-rank p < 0.001), and there was a trend toward improved PFS favoring patients harboring responsive tumors by mWHO criteria (log-rank p = 0.077). These results were not translated into increased OS of responders, according to either criteria (log-rank p = 0.74 and 0.331 for WHO and mWHO, respectively).

**Fig 3:**
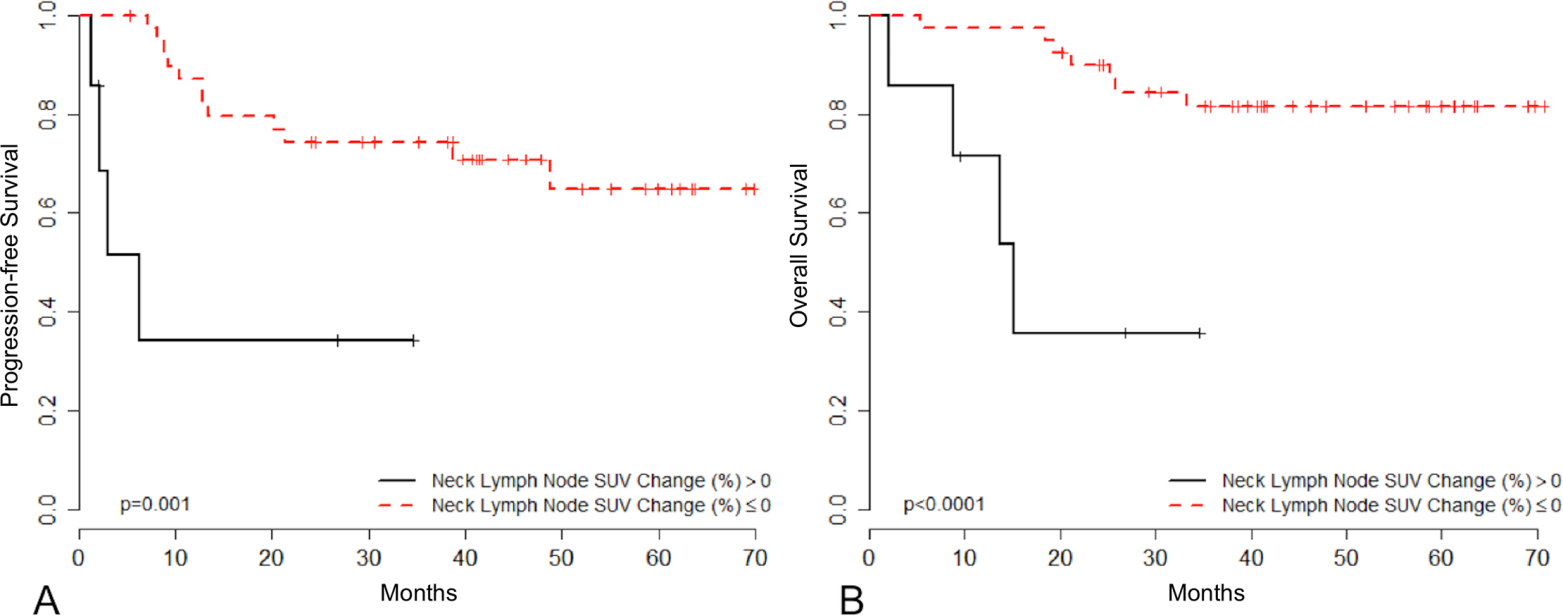
Clinical outcome according to neck lymph node SUV change after cycle 1 of IC. Progression-free survival (3A) and overall survival (3B) of patients who underwent D14-C1-IC ^18^F-FDG PET/CT according to the change in the maximum SUV of regional lymph nodes after the first cycle of IC (N = 47). Patients with tumors demonstrating no change or decrease in neck lymph node SUV (neck lymph node SUV change (%) ≤ 0) were considered responders (N = 40; red) and those with tumors showing an increase in neck uptake (Neck Lymph Node SUV Change (%) > 0) were considered non-responders (N = 7; black).

**Fig 4:**
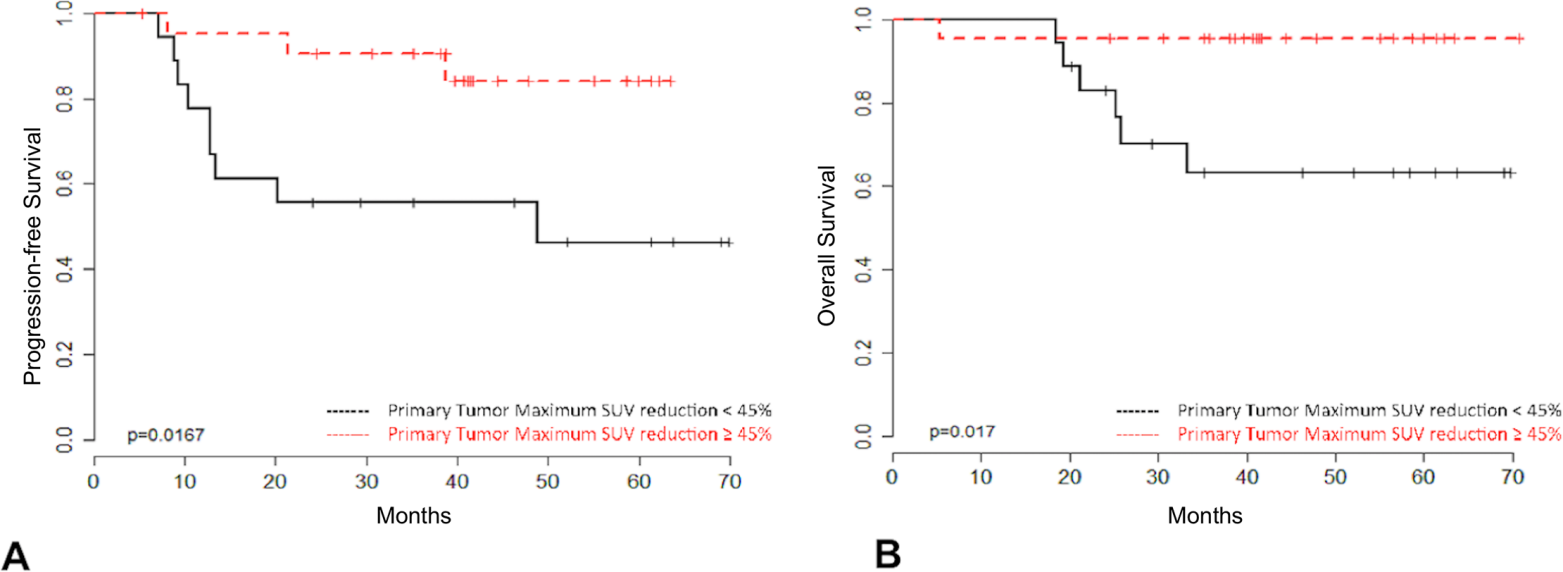
Clinical outcome according to primary tumor SUV extent change after cycle 1 of IC. Progression-free survival (4A) and overall survival (4B) among responsive patients (neck lymph node SUV change (%) ≤ 0) according to the change in maximum primary tumor SUV after the first cycle of IC (N = 40). Patients with maximum primary tumor SUV reduction ≥ 45% (N = 22; red) showed improved outcomes compared with those with primary tumor uptake reduction < 45% (N = 18; black).

Conversely, the 40 patients presenting with D14-C1-IC ^18^F-FDG PET/CT responsive tumors exhibited superior PFS (HR = 0.18, 95% CI 0.056–0.585; p = 0.004) and OS (HR = 0.14, 95% CI 0.040–0.498; p = 0.002) than those with non-responsive tumors (Fig 3). Moreover, among these 40 patients, the extent of decrease in SUV in primary tumors correlated with clinical outcome. According to the maximum of the standardized log-rank statistical analysis, patients whose primary tumors showed an SUV decrease of ≥45% from baseline on D14-C1-IC PET/CT exhibited higher PFS (HR = 0.23, 95% CI 0.062–0.854; p = 0.028) and OS (HR = 0.11, 95% CI 0.013–0.96; p = 0.046) (Fig 4). A post-hoc study power analysis performed using the PFS HR and a two-tailed alpha of 0.05 determined a statistical power of 0.64 (Beta = 0.36) to detect a meaningful difference in PFS according to primary tumor maximum SUV reduction. Bootstrap analysis confirmed these findings for both outcome measures.

**Fig 5:**
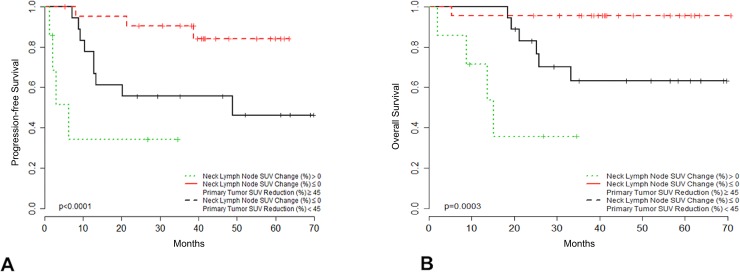
Clinical outcome according to primary tumor/neck lymph node SUV change after cycle 1 of IC. Progression-free survival (5A) and overall survival (5B) among patients according to maximum regional lymph node SUV change and primary tumor SUV change after the first cycle of induction chemotherapy (N = 47). Patients with responsive tumors (neck lymph node SUV change (%) ≤ 0) and maximum primary tumor SUV reduction ≥ 45% (N = 22; red) showed improved outcomes compared with patients with responsive tumors (neck lymph node SUV change (%) ≤ 0) and primary tumor maximum SUV reduction <45% (N = 18; black), or with patients with unresponsive tumors (neck Lymph node SUV change (%) > 0; N = 7; green).

Patients that did not achieve ≥45% decrease in primary tumor SUV and those who experienced an increase in neck SUV, had unfavorable outcomes (PFS and OS) compared with those that had a ≥ 45% reduction in primary tumor SUV associated with any reduction in neck SUV (Fig 5).

### Clinical outcomes of OPSCC patients according to HPV status

Among 39 primary OPSCC patients, 25 (64%) had their tumors tested for HPV. Patients with HPV-positive tumors experienced higher response rates during IC according to both mWHO criteria (100% vs. 86%; p = 0.28) and evaluation of neck lymph node SUV reduction on D14-C1-IC ^18^F-FDG PET/CT (100% vs. 57%; p = 0.015). Patients with HPV-negative tumors had inferior PFS (HR = 4.09, 95% CI 0.818–20.50; p = 0.086), and OS (HR = 9.97, 95% CI 1.03–96.64; p = 0.047). Patients with HPV-positive tumors considered metabolically responsive (no increase in neck SUV) that achieved ≥45% SUV reduction in primary tumors on D14-C1-ICPET/CT (18 patients) experienced excellent outcomes, with no progressive disease or death reported.

## Discussion

Since randomized trials demonstrated the superior efficacy of TPF over PF in LASCCHN, IC followed by CBRT has re-emerged as a therapeutic option; however, TPF- associated toxicities have raised concerns about the feasibility of this approach [41–43]. Even with more powerful IC regimens, 3%–7% and 11%–12% of patients experience disease progression and stability, respectively, and may, therefore, benefit from an early change in therapeutic strategy [10–12]. This prompted a discussion regarding the potential benefit of early evaluation of tumor response, allowing selection of patients with responsive tumors to continue with function preserving approaches, while offering patients with non-responsive tumors alternative salvage treatments. Data regarding the role of early response assessment by conventional methods (endoscopy and CT scan) are scarce. Although early endoscopic examination appears to provide good prognostic information, CT is insufficiently sensitive to detect response at this time [44–46]. Hence, we investigated an alternative response evaluation method, PET/CT, early in the course of treatment.

The use of PET/CT for head and neck cancer management has increased, is considered highly reproducible [47] and presents many indications [31, 32, 48]. Additionally, early metabolic evaluation may be useful during the course of chemotherapy for esophageal cancer, Hodgkin´s lymphoma, and breast cancer [33–36].

The role of metabolic response evaluation at the time of routine tumor assessment (after two cycles of IC) has previously been reported [49–50]. PET/CT could detect distinct subgroups of patients according to the extent of SUV reduction from baseline. This approach has also been tested during IC for LASCCHN after the first cycle of chemotherapy [45, 51–53]. The discovery of metabolic parameters with enhanced prognostic discriminatory capabilities, such as metabolic tumor volume or total lesion glycolysis, has introduced another level of complexity [54–55]. Although it remains unclear which metabolic parameter or threshold is optimal for identification of responsive tumors, the results of these investigations point to a significant prognostic role for early PET/CT during induction chemotherapy. Our group has previously reported findings from a prospective study in which early changes in ADC as measured by MRI was shown to be a potential surrogate for early complete metabolic and morphological 18F-FDG PET/CT response in patients treated with IC for LASCCHN [56].

The present trial demonstrated that patients with an increase in neck SUV after the first cycle of IC experienced inferior outcomes. Furthermore, among those with no elevation in regional lymph node SUV, patients whose primary tumors demonstrated a significant (>45%) decrease in SUV after the first cycle of IC fared better in terms of PFS and OS.

In our study, the prognostic information derived from D14-C1-IC ^18^F-FDG PET/CT was more reliable than that provided by standard morphological criteria (WHO and mWHO). While response according to PET/CT was significantly associated with PFS and OS, the relationship between response and outcomes determined using anatomical criteria was weaker or absent. This is supported by data showing that PET/CT is more accurate for defining clinical and pathological responses, relative to CT and MRI, respectively [45,46,53]. Although response by morphological criteria can predict clinical outcome and response to radiotherapy, according to studies performed before the era of modern imaging techniques, treatment response assessment may be improved by incorporation of newer technologies, such as PET/CT [57, 58].

The high frequency of patients with primary oropharyngeal tumors and the absence of patients with oral cavity cancer in our study population reflect the usual clinical care adopted in our center. Common treatment modalities are IC followed by chemoradiotherapy for OPSCC and immediate surgical resection for oral cavity tumors. The preponderance of HPV-related OPSCC explains the high responsiveness and disease control recorded in our population. The rising incidence of HPV-related OPSCC and its improved prognosis have raised concerns regarding over-treatment related toxicity [22, 59, 60]. Preliminary data from ECOG 1308 showed promising results with low-dose RT, with patients with HPV-positive OPSCC achieving complete response to IC [61]. There is growing interest in developing tools to identify the subgroup of patients for whom treatment de-intensification can be safely applied [19, 62]. In the current trial, patients harboring HPV-related oropharyngeal cancers, whose primary tumors presented SUV decreases of ≥45% experienced impressive outcomes. No progressive disease or death was recorded in this subgroup, suggesting that early PET/CT could be used to select a population for treatment de-intensification.

This study has limitations. First, the criteria used to define tumors as responsive according to regional lymph node SUV change during IC have not previously been validated. Second, among those patients with responsive tumors, the cut-off for prognostication was obtained retrospectively. Third, due to the low number of events in this population, we were unable to independently evaluate the effects of different variables on treatment outcomes. Finally, data regarding HPV status was not available for all oropharyngeal cancer patients. Despite these shortcomings, to the best of our knowledge, this is the largest prospective study assessing the role of early PET/CT during IC (after the first cycle) for head and neck cancer. Additionally, interim PET/CT evaluation was performed in a blinded fashion, avoiding possible interference with patient management. Moreover, patients were treated uniformly according to standard practice, and were recruited within a narrow interval, ensuring consistent, similar patterns of care throughout the study. We think this pilot study provides important information regarding the role of early metabolic tumor-response evaluation for patients with LASCCHN that are treated with IC-CRT. However, we acknowledge our findings do not allow changing the standard clinical practice, as morphological tumor-response evaluation according to the WHO criteria after se second cycle of IC still remains the most validated method to identify patients with tumors sensitive to chemotherapy and radiation therapy. We believe that a practice-changing shift in the management of LASCCHN using metabolic tumor-response assessment during IC would rely on the results of a properly designed randomized controlled trial in which LASCCHN patients treated with IC-CRT would be randomly allocated to undergo tumor-response evaluation by the standard evaluation criteria after the second cycle of IC versus patients undergoing an early 18 FDG PET/CT after the first cycle of IC, and then managed accordingly.

To conclude, D14-C1-IC ^18^F-FDG PET/CT evaluation is a promising tool to identify patients with responsive tumors during IC. Further studies are necessary to confirm these findings, and explore their implications for patient management. Additionally, at a time when de-intensification of HPV-related oropharyngeal cancer is proposed, early PET/CT provides a potential tool to select those patients with tumors responsive to less aggressive treatment strategies.

## Supporting information

S1 Table Patients and tumor characteristics. (DOCX)

S2 Table Detailed description of staging and treatment. This is the S2 table legend D: Docetaxel; P: Paclitaxel; C: Cisplatin; Ca: Carboplatin; F: 5-Fluoracil; Cx: Cetuximab; N/A1: not applicable (toxic death after Cycle 3 Induction Chemotherapy); N/A2 not applicable (exclusive radiotherapy post-Induction Chemotherapy).
(DOCX)

S1 File This is ethic committee. (DOCX)

S2 File Comitê de Ética: This is renamed_ffc93. (DOCX)

S3 File TREND statement checklist: This is TREND statement checklist. (DOCX)
