# Supplementary material for: Early metabolic 18F-FDG PET/CT response of locally advanced squamous-cell carcinoma of head and neck to induction chemotherapy: A prospective pilot study
Source: PLoS One. 2018 Aug 16;13(8):e0200823. doi: 10.1371/journal.pone.0200823 (PMC6095513; doi:10.1371/journal.pone.0200823)
Supplement: S1 Table — (DOCX) [file pone.0200823.s001.docx]

**Supplementary Table 1.** Patients and tumor characteristics.

| **Patient no.** | **Sex** | **Age** | **Tumor site** | **Clinical stage** | **Smoking history** | **HPV status** |
| --- | --- | --- | --- | --- | --- | --- |
| **1** | male | 39 | oropharynx | IVA | current | positive |
| **2** | male | 74 | oropharynx | IVA | current | unknown |
| **3** | male | 46 | oropharynx | IVA | current | positive |
| **4** | male | 40 | oropharynx | IVA | current | positive |
| **5** | male | 57 | hypopharynx | IVB | current | unknown |
| **6** | male | 58 | oropharynx | IVA | current | positive |
| **7** | male | 45 | larynx | III | former | unknown |
| **8** | male | 44 | oropharynx | III | current | positive |
| **9** | male | 48 | oropharynx | IVB | former | positive |
| **10** | male | 66 | oropharynx | IVA | never | positive |
| **11** | male | 50 | oropharynx | III | never | positive |
| **12** | male | 43 | oropharynx | IVA | never | positive |
| **13** | male | 59 | hypopharynx | IVA | current | unknown |
| **14** | female | 71 | hypopharynx | IVA | current | unknown |
| **15** | male | 59 | larynx | IVA | current | unknown |
| **16** | male | 47 | oropharynx | IVA | current | unknown |
| **17** | male | 55 | oropharynx | IVA | current | negative |
| **18** | male | 50 | oropharynx | IVA | former | unknown |
| **19** | male | 60 | oropharynx | III | former | unknown |
| **20** | female | 48 | larynx | IVA | former | unknown |
| **21** | male | 59 | oropharynx | IVA | current | unknown |
| **22** | male | 65 | oropharynx | IVA | never | positive |
| **23** | male | 55 | oropharynx | IVA | never | unknown |
| **24** | male | 61 | oropharynx | IVA | former | positive |
| **25** | male | 57 | oropharynx | IVA | current | negative |
| **26** | male | 58 | oropharynx | IVA | current | negative |
| **27** | male | 52 | oropharynx | III | never | unknown |
| **28** | male | 61 | oropharynx | IVB | former | unknown |
| **29** | male | 46 | oropharynx | IVB | never | unknown |
| **30** | female | 56 | oropharynx | IVA | former | unknown |
| **31** | male | 68 | oropharynx | III | current | negative |
| **32** | male | 46 | oropharynx | III | former | positive |
| **33** | male | 53 | oropharynx | IVA | never | positive |
| **34** | male | 53 | oropharynx | IVB | former | unknown |
| **35** | male | 54 | oropharynx | IVB | former | unknown |
| **36** | male | 45 | oropharynx | IVB | never | positive |
| **37** | female | 66 | oropharynx | III | former | unknown |
| **38** | male | 63 | oropharynx | IVA | former | positive |
| **39** | male | 51 | oropharynx | IVA | current | unknown |
| **40** | male | 50 | oropharynx | IVB | current | negative |
| **41** | male | 51 | hypopharynx | III | current | unknown |
| **42** | male | 58 | larynx | IVB | former | unknown |
| **43** | female | 54 | oropharynx | IVA | never | negative |
| **44** | male | 57 | oropharynx | IVA | never | positive |
| **45** | male | 56 | oropharynx | IVB | never | positive |
| **46** | male | 65 | oropharynx | IVA | former | negative |
| **47** | male | 44 | oropharynx | IVA | never | positive |
| **48** | male | 67 | oropharynx | IVA | current | unknown |
| **49** | male | 52 | oropharynx | IVA | never | positive |
