# Supplementary material for: Early metabolic 18F-FDG PET/CT response of locally advanced squamous-cell carcinoma of head and neck to induction chemotherapy: A prospective pilot study
Source: PLoS One. 2018 Aug 16;13(8):e0200823. doi: 10.1371/journal.pone.0200823 (PMC6095513; doi:10.1371/journal.pone.0200823)
Supplement: S2 Table — This is the S2 table legend D: Docetaxel; P: Paclitaxel; C: Cisplatin; Ca: Carboplatin; F: 5-Fluoracil; Cx: Cetuximab; N/A1: not applicable (toxic death after Cycle 3 Induction Chemotherapy); N/A2 not applicable (exclusive radiotherapy post-Induction Chemotherapy). (DOCX) [file pone.0200823.s002.docx]

**Supplementary Table 2.** Detailed description of staging and treatment.

| **Patient no.** | **T** | **N** | **Induction chemotherapy** | **Radiotherapy** | **Concurrent CT regimen** | **Salvage surgery** |
| --- | --- | --- | --- | --- | --- | --- |
| **1** | 4a | 2a | DCF | IMRT 70 Gy | Ca | no |
| **2** | 4a | 2b | DCF | 3D 70.4 Gy | Cx | no |
| **3** | 2 | 2a | DCF | 2D 70.4 Gy | Ca | no |
| **4** | 4a | 2b | DCF | 3D 70.4 Gy | Ca | yes |
| **5** | 3 | 3 | DCF | IMRT 70 Gy | C | yes |
| **6** | 3 | 2c | DCF | IMRT 70 Gy | C | no |
| **7** | 3 | 1 | DCF | 3D 70.4 Gy | Ca | no |
| **8** | 3 | 0 | DCF | IMRT 70 Gy | Ca | no |
| **9** | 2 | 3 | DCF | IMRT 70 Gy | Ca | no |
| **10** | 2 | 2b | DCF | IMRT 70 Gy | Ca | no |
| **11** | 2 | 1 | DCF | IMRT 70 Gy | Ca | no |
| **12** | 3 | 2b | DCF | IMRT | C | no |
| **13** | 2 | 2b | DCF | IMRT 70 Gy | C | no |
| **14** | 3 | 2b | DCF | 3D 70.4 | Ca | yes |
| **15** | 3 | 2c | DCF | 3D 70.2 Gy | Ca | yes |
| **16** | 4a | 2c | PCF | 2D 70.4 Gy | Ca | no |
| **17** | 3 | 2b | DCF | N/A1 | N/A1 | yes |
| **18** | 4a | 2b | DCF | N/A1 | N/A1 | no |
| **19** | 3 | 1 | DCF | IMRT 70 Gy | Ca | no |
| **20** | 1 | 2a | DCF | 3D 70.4 Gy | Ca | no |
| **21** | 3 | 2a | DCF | IMRT 70 Gy | Ca | no |
| **22** | 3 | 2b | DCF | 3D 70.4 Gy | Ca | no |
| **23** | 2 | 2b | DCF | 3D 70.4 Gy | Ca | no |
| **24** | 2 | 2c | DCF | IMRT 70 Gy | Ca | yes |
| **25** | 3 | 2c | DCF | IMRT 70 Gy | Ca | yes |
| **26** | 1 | 2c | DCF | 3D 70.4 Gy | N/A2 | no |
| **27** | 3 | 1 | PCF | 3D 70.4 Gy | C | no |
| **28** | 4b | 1 | PCF | 3D 70.4 Gy | Ca | no |
| **29** | 4b | 0 | DCF | IMRT 70 Gy | Ca | no |
| **30** | 4a | 2c | DCF | IMRT 70 Gy | Ca | no |
| **31** | 3 | 1 | DCF | IMRT 70 Gy | Ca | no |
| **32** | 3 | 1 | DCF | IMRT 70 Gy | Ca | no |
| **33** | 3 | 2b | DCF | IMRT 70 Gy | Ca | no |
| **34** | 4b | 2b | PCF | 3D 70,4 Gy | C | yes |
| **35** | 2 | 3 | PCF | 3D 70.4 Gy | Ca | yes |
| **36** | 3 | 3 | DCF | IMRT 70 Gy | Ca | no |
| **37** | 3 | 1 | DCF | IMRT 70 Gy | Ca | no |
| **38** | 2 | 2b | DCF | IMRT 70 Gy | Ca | no |
| **39** | 4a | 2b | DCF | IMRT 70 Gy | Ca | yes |
| **40** | 2 | 3 | DCF | IMRT 70 Gy | Ca | yes |
| **41** | 3 | 0 | PCF | IMRT 70 Gy | Ca | no |
| **42** | x | 3 | DCF | 3D 70.4 Gy | Ca | no |
| **43** | 2 | 2b | DCF | IMRT 70 Gy | Ca | no |
| **44** | 2 | 2a | DCF | IMRT 70 Gy | Ca | no |
| **45** | 2 | 3 | DCF | IMRT 70 Gy | Ca | no |
| **46** | 3 | 2c | DCF | IMRT 70 Gy | Ca | no |
| **47** | 1 | 2b | DCF | IMRT 70 Gy | Ca | no |
| **48** | 3 | 2b | DCF | IMRT 70 Gy | Cx | yes |
| **49** | 3 | 2c | DCF | IMRT 70 Gy | Cx | no |

D: Docetaxel; P: Paclitaxel; C: Cisplatin; Ca: Carboplatin; F: 5-Fluoracil; Cx: Cetuximab; N/A1: not applicable (toxic death after Cycle 3 Induction Chemotherapy); N/A2 not applicable (exclusive radiotherapy post-Induction Chemotherapy)
