# Supplementary material for: Early metabolic 18F-FDG PET/CT response of locally advanced squamous-cell carcinoma of head and neck to induction chemotherapy: A prospective pilot study
Source: PLoS One. 2018 Aug 16;13(8):e0200823. doi: 10.1371/journal.pone.0200823 (PMC6095513; doi:10.1371/journal.pone.0200823)
Supplement: S1 File — (DOCX) [file pone.0200823.s003.docx]

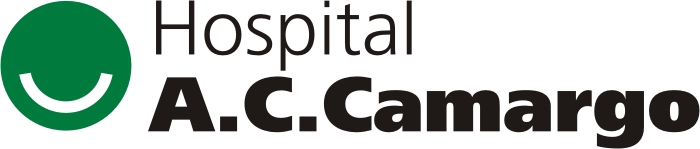


18.FDG PET-CT USE AS AN EFFECTIVE PREDICTOR FOR LOCOREGIONAL TUMOR CONTROL AND SURVIVAL IN CARCINOMA EPIDERMÓIDE OF HEAD AND NECK.

**Principal Investigator:**

Ulisses Ribaldo Nicolau

**Sub Investigators:**

Tadeu Ferreira de Paiva Jr

Paula Nicole Vieira Pinto

Antonio Cássio de Assis Pellizzon

Marcello Ferretti Fanelli

Eduardo Nóbrega Pereira Lima

Luiz Paulo Kowalski

São Paulo, july, 2009

Introduction

 Head and neck squamous cell carcinoma (SCCHN) accounts for 5% of newly diagnosed neoplasms, 644,000 cases and 350,000 deaths per year worldwide (1). In this anatomical location, tumors occur more frequently in the oral cavity (44%), larynx (31%) and pharynx (25%). The majority of cases present as potentially curable locally advanced tumors. Distant metastases to diagnosis occur in up to about 10% of cases. Despite advances in treatment, disease-free survival (DFS) and overall survival (OS) remain unsatisfactory. About 40% to 60% and 20% to 30% of patients will have local/ regional and systemic relapse respectively. Among the factors implicated in the prognosis of these patients, the staging, clinical condition of the patient and factors such as smoking, alcohol use and HPV infection in oropharyngeal tumors (identifying a group of patients carrying a better prognosis) are established as pretreatment factors influencing clinical outcome (2, 3,4).

The treatment of head and neck tumors is highly complex and varies according to different anatomical sites, the difficulty of access to these sites and the importance of preserving organs and structures. In general, patients with early tumors are treated with surgery or radiotherapy, while those with advanced tumors receive combined therapies, associating in varied sequences surgery radiotherapy and chemotherapy. In recent years, chemotherapy has assumed a greater role in the treatment of locally advanced disease, being used in different strategies: induction (previously) , and concurrent to radiotherapy. The preservation of organs with clinical treatments by the combination of chemotherapy and radiotherapy, reserving surgical resection to the non-responders is part of the clinical practice, however, in spite a great debate about the ideal timing of the association between chemotherapy and radiotherapy (induction versus concurrent association) capable of achieve better results in terms of local control and survival. Rigorous patient selection, monitoring of side effects, and methods to evaluate patients responsive to treatment are important in order to minimize toxicity.

Positron emission tomography is a imaging method used as part of diagnostic, staging and evaluation of response to neoplasms in increasing use in clinical practice. Among the isotopes in use and in research, the use of radiotracer 18-F-fluorodeoxyglucose (18-F-FDG) has increased recently. Considering the possibilities of benefit of the use of PET-CT in squamous-cell carcinoma of the head and neck, it is highlighted its potential role the planning of radiotherapy, as well as subsequent therapeutic decision-making regarding cervical emptying and approach of the primary tumor at the end of the treatment radiotherapy, issues that are gaining relevance due to the increasing use of radiotherapy associated with chemotherapy in this entity.

It is also observed the potential use of this method for early evaluation of established therapies. When early prediction of the efficacy of therapies of high toxicity at the onset of these therapies, the benefit of selecting a fraction of patients that can be spared from treatments that lead to morbidity, with the possibility of early switching to alternative therapies, is sought.

**Historic and background**

1- Association of radiotherapy and chemotherapy in Squamous-Cell Carcinoma of Head and Neck

During the 1990s several studies evaluated the association of chemotherapy concomitant with radiotherapy, demonstrating greater efficacy in locoregional control and consequent possible impact on DFS and OS (5,6,7).

Jeremic et al. Randomized 130 patients with a diagnosis of locally advancedSCCHN for phase III study containing 02 arms, the control arm composed of patients submitted to hyperfractionated radiotherapy, 77 Gy, versus the experimental arm in which patients were submitted to the same radiotherapy regimen associated with daily cisplatin , 6mg / m2. Overall survival of 68% versus 49% at 2 years, and 46% versus 25% at 5 years (p = 0.0075) was achieved for the arm who underwent combined treatment versus exclusive radiotherapy. Significant gains in progression-free survival, regional locus-free progression-free survival and suppression of distant metastasis were also observed in the group receiving concomitant combined treatment5.

Corroborating with findings coming from additional studies that demonstrated an advantage for local control and survival for the chemotherapy modality concomitant with radiotherapy, a meta-analysis published by Pignon et al. In 2000 showed benefit in the combination of chemotherapy and local treatment for locally advanced SCCHN.

Sixty three studies, containing 10 741 patients submitted to locoregional treatment with or without chemotherapy, showed a significant survival gain of 4% in 2 and 5 years in favor of the combination of chemotherapy (7).

Larynx

 In 1991, an important trial was published evaluating the use of induction chemotherapy, consisting of cisplatin associated with continuous infusion of 5-fluorouracil, followed by radiotherapy in the responsive patients; with the control arm being the standard treatment, consisting of total laryngectomy followed by adjuvant radiotherapy.

Among some important findings, a 64% rate of laryngeal preservation was observed, with no decrease in survival in the group of patients treated with induction chemotherapy followed by radiotherapy when compared to the control arm (2-year survival rate of 68% in both groups). Furthermore, 85% of patients presented responsive tumors after 02 cycles of chemotherapy, with 31% rate of complete tumor response(8).

In 2003, Forastiere et al. published a trial in which 3 therapy strategies were evaluated, including, induction chemotherapy versus the concomitant combination of cisplatin and radiotherapy versus radiotherapy exclusively for patients with locally advanced laryngeal SCC, candidates for total laryngectomy with curative intent. Despite a similar overall survival in the three groups, the concomitant combination of chemotherapy and radiotherapy was superior to induction chemotherapy or exclusive radiotherapy for locoregional control and preservation of the larynx. Ana trend of lower recurrence rate was observed for the 02 groups that received chemotherapy in comparisson to the arm that received isolated radiotherapy (9).

Hypopharynx

Lefebvre and colleagues reported a trial in which patients harboring locally advanced hypopharyngeal squamous cell carcinoma candidates to radical pharyngolaryngectomy with curative intent were randomized to induction chemotherapy followed by radiotherapy in responsive patients, in the same way as the study carried out by the Veterans Affairs Latyngeal Cancer Study Group (experimental arm), or surgical resection by total pharyngolaryngectomy followed by radiotherapy (control arm).The group of patients who underwent induction chemotherapy obtained a complete response rate of 54% in the primary tumor and 51% in cervical lymph nodes, in addition to achieve a organ preservation rate in 3 and 5 years of 42% and 35%, respectively. There was no difference in locoregional recurrence when compared to the surgical group. There was also a trend for a lower rate of distant recurrence in the group submitted to induction chemotherapy, with no significant difference in overall survival (10).

Oropharynx

Domenge and colleagues evaluated the role of induction chemotherapy with cisplatin and 5-fluorouracil followed by a locoregional approach consisting of surgery followed by radiotherapy or exclusive radiotherapy, versus the control arm consisting of treatment consisting of surgery followed by radiotherapy for patients diagnosed with Locally advanced oropharynx SCC that can be treated by surgery or radiotherapy. The trial randomized 318 patients and a better median survival of the group treated with induction chemotherapy was observed in comparison with patients receiving no chemotherapy, with a median survival of 5.1 years and 3.3 years (p = 0.03), respectively (11).

Calais et al. evaluated the addition of carboplatin and 5-fluorouracil concurrent to radiotherapy compared to the the control arm that received radiotherapy exclusively in patients with locally advanced oropharyngeal SCC.

A total of 266 patients were treated, with results demonstrating better outcome favoring combined chemo radiotherapy over exclusive radiotherapy, with 5 yearsOS of 22% versus 16%, p = 0.05, respectively. Disease-free survival (27% versus 15%, p = 0.01) and locoregional control (48% versus 25% p = 0.002) also favored the group receiving combined treatment (12).

2- Induction chemotherapy

In the 1990s, induction chemotherapy followed by radiotherapy was established as an optional treatment feasible for patients with locally advanced head and neck SCC candidates for organ preservation. Despite the potential benefits of this strategy, including potential for suppression of distant metastasis, such association failed to achieve overall survival benefit when compared to exclusive radiotherapy or radiotherapy concomitant with chemotherapy.

In view of the combined concomitant use of radiotherapy with chemotherapy in the last two decades, induction chemotherapy has again been discussed by the emergence of new agents such as taxanes, with increasing interest in the association of such chemotherapeutics with previously used regimens, in order to explore its potential therapeutic benefits , such as higher response rates, suppression of distant metastases and potential impact on survival.

Among the various agents, the association of docetaxel or paclitaxel with cisplatin and 5-fluorouracil is highlighted.

Hitt et al, in 2005, studied 382 patients with resectable or unresectable head and neck squamous-cell carcinoma for treatment with induction chemotherapy with cisplatin 100mg / m² day1 and 5FU 1000 mg / m² day 1 to day 5 versus cisplatin 100mg / m² day 1, 5FU 500mg / m² D2 to D6 and paclitaxel 175mg / m² D1, applied every 21 days for 3 cycles. In both arms, patients with responsive tumors underwent radiotherapy receiving 70Gy radiation therapy concurrent with Cisplatin 100mg / m² D1, D22 and D43.

Patients treated in the paclitaxel containing induction chemotherapy group presented a higher rate of complete response (33% vs 14%, p <0.001), in addition to a trend towards better OS (43 months vs 37 months, p = 0.06) (13).

A phase I / II study published in 2004 evaluated the use of docetaxel, cisplatin and 5-fluorouracil at two dose levels. Patients were evaluated for two cisplatin dosages ( 100 mg / m2 or 75mg/m2) one day and docetaxel 75 mg / m² one day associated with 5-Fluorouracil 750 mg / m2 / 24h continuous infusion for 96 hours. There was a better toxicity profile in the scheme that used cisplatin 75mg / m², in addition to a lower incidence of febrile neutropenia after the inclusion of prophylactic ciprofloxacin(14).

Based on the prior study, docetaxel, cisplatin and 5-fluorouracil (TPF) was evaluated by Vermorken and coworkers that compared to cisplatin, 5-fluorouracil combination (FP) as induction chemotherapy followed by radiotherapy for locally advanced, unresectable head and neck SCC.. A surgical approach to the primary tumor was reserved if residual post-radiotherapy disease was observed, as well as cervical neck dissection for N2-3 lesions regardless of therapeutic response or residual disease.

A total of 358 patients, 177 in the arm that received TPF and 181 in the arm that received PF for 4 cycles prior to radiotherapy, the arm with 03 drugs presented gains in tumor progression free survival (11.0 months versus 8.2 months, p = = .007), also translating into a significant overall survival gain for the arm receiving induction chemotherapy with taxane containing medication (median OS: 18.8 months versus 14.5 months, p = 0.02) (15).

In a randomized prospective study, published by Posner et al., it was demonstrated that induction chemotherapy using docectaxel with PF as compared to PF, followed by radiation associated with radiosensitizing weekly carboplatin was more efficient in terms of OS 3a (49% vs 37%, p = 0.004) and SG 3a (62% vs 48%, p = 0.006). Most patients had locally advanced oropharynx SCC (53%), followed by larynx (17%), oral cavity (15%) and hypopharynx (14%) sites. Thirty-five percent were considered unresectable and 65% resectable (31% considered low curability and 34% with a view to organ preservation).

In a trial recruiting 295 patients (a planned sample of 386 patients) with locally advanced, unresectable, head and neck SCCwas presented by Hitt et al at ASCO 2006, in which induction taxane plus PF was compared with PF followed radiotherapy concurrent to cisplatin, and a third study arm, which consisted of the exclusive concurrent combination of cisplatin and radiotherapy, an higher efficacy was recorded for patients treated with induction chemotherapy, mostly for the taxane containing arm. Patients receiving induction chemotherapy achieved a complete response of 70% versus 49% for patients who were randomized to exclusive concurrent chemoradiotherapy, leading to a progression-free survival gain for induction chemotherapy, more important for the TPF regimen (16 months) against PF (12 months) followed by chemoradiotherapy, to the PFS of 8 months for exclusive chemoradiotherapy (p = 0.002) (17).

 The study published by Hitt et al. therefore raised the discussion of the possible replacement of the concurrent use of chemotherapy and radiotherapy by the use of induction chemotherapy followed by concurrent chemotherapy and radiotherapy in patients harboring locally advanced SCC of head and neck.

The above mentioned data show the tendency in many centers to use docetaxel with cisplatin and 5-fluorouracil as an induction regimen, which is based on the studies called TAX 323 and TAX 32418.

Evaluation of induction chemotherapy tumor- response in Squamous-cell carcinoma of the head and neck

In accordance with other solid tumors, squamous cell carcinoma of the head and neck has evaluated tumor response to chemotherapy and radiotherapy by criteria such as RECIST (19), with one-dimensional measurement of lesions and post-treatment comparisons, or by World Health Organization (WHO) criteria (20) and their variants. Thus, a recent study conducted by Posner et al (16) used the modified WHO criteria (16), whose peculiarity was to consider a partial response (PR) decrease of 25% or more of the product of the largest diameters of the measurable lesions. Imaging findings (computed tomography or magnetic resonance imaging) were therefore considered for the primary tumor and neck lymph node, as well as findings by complementary tumor evaluation specific for tumors in this location (eg nasofibrolaryngoscopy). It is worth observing the criterion most frequently used in these studies, which considers a partial response reduction of at least 50% in the product of the measurable lesions (20). Thus, in the second cycle of induction chemotherapy, those who are considered carrying tumors considered chemo-responsive would undergo the third cycle of chemotherapy and subsequent association of radiotherapy and concomitant chemotherapy. It is also observed that the criteriafor considering a patient as carrying a chemo-responsive tumor to the second cycle and the assumption of radiosensitivity by this criteria guided most of the studies of induction chemotherapy in the last two decades. Thus, the association of radiotherapy to chemotherapy followed by discussion of the subsequent surgical approach to the primary tumor and neck depends on TNM staging and subsequent evaluation by the tumor response according to the WHO or RECIST criteria, which are based on imaging findings and are complemented by clinical (physical exam) and laboratory evaluations. Despite this clinical approach having an established role in the clinical practice for treating patients in stages III and IV, high rates of relapse and the existence of a fraction of patients harboring non-responsive tumors, added to the relevant clinical toxicity associated with this approach, there is a potential role of establishing an early assessment of tumor chemo and radiotherapy responsiveness for treated patients.

Selection at the beginning of treatment of patients with a low probability of responding to chemoradiotherapy treatment allows them to discuss their therapeutic strategy aiming at an early re-evaluation of resectability, with the benefit of not exposing them to treatment with high toxicity and low probability of success. In this scenario, noninvasive tests that associate functional characteristics have the potential benefit of early evaluation of the efficacy of ongoing therapies. The main failures considered by PET in the assessment of tumors are the absence of anatomical markers and the difficulty of differentiating inflammatory activity, which also incorporates FDG, of tumor activity (21). However, recently the PET-coupled PET prototype was introduced allowing the collection of co-registered PET and CT images in a single examination session(22). The combination of these modalities improved the identification and definition of the abnormalities identified by the FDG-PET and the association of biological and anatomical information offered a better definition of the tissue abnormalities (23).

Retrospective analyzes and prospective studies attempt to establish the role of FDG-PET in staging of squamous cell carcinoma of the head and neck. In centers that have this equipment , its use in the follow-up of these patients after treatment of the primary tumor increases, usually after 2 to 3 months after the end of the radiotherapy. This is an opportunity to discuss the cervical approach of initially positive patients who present a response after radiotherapy and chemotherapy, in addition to evaluating the primary tumor.

Considering the role established for early evaluation in other tumors by FDG-PET, Kostakoglu et. al evaluated 47 patients diagnosed with diffuse large B-cell lymphoma and Hodgkin's lymphoma prospectively by performing a pre-examination and early re-evaluation with new FDG-PET after 1-cycle chemotherapy. Among the 31 patients with negative results to the FDG-PET performed after the first cycle, all presented a sustained response confirmed after a follow-up of 28 months. Among the 16 patients with a positive test (non-complete response) after the first cycle, 14 presented refractory disease or tumor recurrence in the period (24).

**Objective**

To evaluate the role of SUV drop between dedicated 18F-FDG PET-CT prior to initiation of treatment after first cycle induction chemotherapy (at D14) and after termination of chemoradiotherapy treatment in patients with squamous cell carcinoma of the head and neck stages III or IV resectable or nonresectable non-metastatic patients treated with neoadjuvant chemotherapy followed by radiotherapy or chemoradiotherapy. The findings in PET CT will be correlated with the response rate after the 2nd cycle of chemotherapy after chemoradiotherapy, locoregional control, progression-free survival and overall survival, which will be determined by standard methods such as clinical examination, endoscopy (if indicated ) and by Computed Tomography.

It should be noted that clinical decisions will be made according to methods established in the current treatment policy, such as those observed by WHO response criteria. Thus, data regarding PET-CT findings will not be considered in the evaluation of response and decision in the continuation of chemotherapy and radiotherapy.

Study Population

This is an uncontrolled and unicentric prospective study that will be performed at the Hospital AC Camargo in the Departments of Image, Clinical Oncology, Head and Neck Surgery and Radiotherapy. Sixty patients with a diagnosis of squamous cell carcinoma of the head and neck, non-metastatic stages III and IV, without previous treatment for neoplasia, will be recruited.

**Patients and Methods**

 Patients will be diagnosed and staged by imaging methods and and physical examination according to current conventional practice, as described below. Up to 10 days before the start of the first cycle of chemotherapy, in 14th day of cycle 1 (with a tolerance ranging from 12th to 16th day induction chemotherapy-cycle 1). ). A third PET-CT will be performed 8 to 12 weeks after the end of radiotherapy if the assistant physician judge it as useful for tumor evaluation. It is observed that the investigators of the specialties clinical oncology, head and neck surgery and radiotherapy will be blinded to the results of the baseline and second (D14 cycle 1) PET-CT performed, which will be evaluated only by the image department investigator. At this time, as part of the pre-screening screening, the investigator located in the Imaging Department should provide information to other researchers regarding the absence of any suspicion of distant metastatic dissemination for each patient evaluated. If any investigator is suspected of the possibility of distant metastasis, information regarding the first PET-CT, associated with the other tests performed, should be confronted. Thus, the criteria for evaluation of tumor-response and therapeutic decisions until the end of radiotherapy will not influence the findings of the first or the second PET-CT performed. Clinical decisions should be based to date on methods used in clinical practice (clinical examination, endoscopic examination if indicated, and computed tomography). An option for a third PET-CT will be performed for patients after the end of radiotherapy as part of imaging evaluation ,and other physicians and researchers will have access to it to aid in the therapeutic decision during the follow-up for decisions regarding the clinical approach of the primary tumor cervical lymph nodes.

Inclusion criteria

Patients may be included in the study if they meet all of the following eligibility criteria:

a- Histological diagnosis of primitive squamous cell carcinoma of the oropharynx, hypopharynx, larynx.

b- Histological diagnosis of early oral cavity epidermoid carcinoma considered to be unresectable.

c- Age range between 18 and 70 years.

d- Tumors of stages T2 (N +) to T4, N0 to N3, M0.

- Candidates for radical surgical treatment for radically resectable locoregional disease.

f- Patients with advanced locoregional disease that is unresectable, without distant metastasis.

During initial evaluation, the surgeon should register resectability (discriminate if the patient presents a tumor considered resectable or unresectable).

g- Clinical conditions that allow both radical surgery (ASA I to III) and neoadjuvant chemotherapy with docetaxel, cisplatin and 5-fluorouracil or radiotherapy associated with platinum chemotherapy.

h- Absence of previous treatments of the tumor as surgery, radiotherapy or chemotherapy. Prior biopsy of the lesion and tracheostomies will be allowed.

i- Performance condition (Karnofsky scale) of 70 or more.

j- Estimated life expectancy of at least 6 months.

k- Adequate marrow reserve: leukocytes> 3,500 / mm3, neutrophils> 1,500 / mm3, platelets> 100,000 / mm3; hemoglobin> 9.0 g / dl.

l- Adequate hepatic function: total bilirubin <1.5 times the upper limit of normal; TGO and TGP <3 times the upper limit of normal.

m- Adequate renal function: creatinine <1.5 times the upper limit of normal, creatinine clearance 60ml / 1.73m2 / 24h or greater.

n- Patient adherence and geographical proximity that allow adequate follow-up.

o- Acceptance to participate in the study as a volunteer and signing of informed consent.

Exclusion Criteria

a- Heart disease active or not compensated by treatment, or acute myocardial infarction in the previous 6 months.

b- Active infection present (at the discretion of the investigator).

c- Concomitant systemic diseases considered serious by the investigator.

d - Another primary neoplasm (except in situ carcinoma of the uterine cervix or adequately treated basal cell carcinoma of the skin), except after curative treatment (without radiotherapy or cervico-facial surgery) for more than 5 years.

- Presence of severe psychiatric illness.

f- Participation in another experimental drug protocol

Pre-study selection

a- Obtaining Informed Patient Consent prior to study entry.

b- History and physical examination (weight, height, performance condition (Karnofsky), vital signs).

c- Hematological examination (Hemogram with platelets)

d- Biochemistry (Na, K, Mg, Urea, creatinine, Ca, albumin, alkaline phosphatase, TGO, TGP, LDH, bilirubin, urinalysis, pregnancy test for women); creatinine clearance (creatinine clearance 60ml / 1.73m2 / 24h or greater).

- e Computed tomography or cervical magnetic resonance. Chest X-ray for cases with staged neck as N0 or N1 and Chest CT for staging with N2 or N3.

 At this time, conventional tests used in patient staging will be analyzed by the researchers. The initial PET-CT scan will be evaluated by the imaging investigator, and the patient will provide information regarding their findings only if there is suspicion of distant metastasis. Likewise, any suspicion of distant metastases by any of the investigators will lead to the access of all investigators to the finding of the first PET-CT.

f- Loco-regional head and neck examination.

g- Biopsy confirming the histopathological diagnosis of epidermoid carcinoma of the head and neck.

**Treatment**

Patients will receive chemotherapy treatment associated with radiotherapy in the neoadjuvant mode, with induction chemotherapy being one of the regimens frequently used in clinical practice according to available evidence for this therapeutic modality, which associates taxanes (paclitaxel or docetaxel) with cisplatin and cisplatin. infusional fluorouracil, followed by radiotherapy alone or in combination with platinum-based chemotherapy, according to the examples listed below:

1- Paclitaxel 175mg / m² EV D1, Cisplatin 100mg / m² EV D1 and 5FU 500mg / m² EV continuous infusion D2 to D6. The chemotherapy regimen will be repeated every 3 weeks for 3 cycles13.

2- Docetaxel 75 mg / m2 EV D1, Cisplatin 75 mg / m2 EV D1 and 5FU 750mg / m2 EV continuous infusion D1 to D5. Prophylactic antibiotic therapy: Ciprofloxacin 500mg every 12 hours for 10 consecutive days, started on D5. The chemotherapy regimen will be repeated every 3 weeks for up to 4 cycles15.

3- Docetaxel 75 mg / m2 EV D1, Cisplatin 100 mg / m2 EV D1 and 5FU 1000mg / m2 EV in continuous infusion D1 to D4. Prophylactic antibiotic therapy: Ciprofloxacin 500mg every 12 hours for 10 consecutive days, started on D5. The chemotherapy regimen will be repeated every 3 weeks for 3 cycles 16.

Granulocyte colony stimulating factors will be used if febrile neutropenia or neutropenia is maintained in Day 28.

**Response evaluation**

In the second cycle, patients will be submitted to imaging response according to modified World Health Organization (mWHO) and World Health Organization criteria, as shown below. Patients who present at least a partial response with a reduction of at least 25% in the product of the largest diameters of the target lesions should receive the entire course of therapy, which comprises a total of 3 or 4 cycles depending on protocols used in clinical practice, followed of local treatment with radiation therapy with or without radiosensitizing platinum.

**Radiotherapy**

70 Gy will be given as total dose of radiotherapy, 1.8 Gy in single daily fraction for 5 days / week, for 7 and 1/2 weeks. The treatment will be performed in a 4 MeV linear accelerator in fields encompassing the cervicofacial region up to 50.4 Gy, with medullary protection at 45 Gy, followed by 2 reductions on the primary lesion at 50.4 and 64 Gy. The final boost, after 64 Gy, should encompass the primary lesion with margins of 1 cm.

For administration of radiotherapy the patient will be immobilized with a mask in the supine position. Cervical nodules, when present, should be identified with lead. Previous fields will be used for the treatment of the inferior cervical region and supraclavicular fossae and opposing lateral fields for treatment of the primary tumor.

Patients will receive, at the discretion of the clinical oncologist, one of the following options:

1- Isolated radiotherapy

2- Radiation therapy associated with Carboplatin AUC 1.5 [Dose in mg = Calculated CLcr + 25) x1,5] weekly, for a maximum of 7 weeks.

Radiation therapy associated with Cisplatin 100mg / m² D1, D22 and D43.

**Surgery**

All patients enrolled in the study should undergo a medical consultation with a head and neck a surgeon prior to the start of treatment, which should consist of:

- Loco-regional examination of the head and neck, including examination of the oral cavity, oropharynx, indirect laryngoscopy or laryngofibroscopy and neck palpation.

- Computed tomography or magnetic resonance imaging of the pharynx and neck without and with contrast.

This should be done within 4 weeks of starting treatment.

Patients should be reassessed with clinical examination, nasopharyngoscopy, and tomography or resonance after no more than 2 weeks after the last application of 2 cycles of induction chemotherapy in the fifth week of treatment, and 6 to 12 weeks after the end of radiotherapy. Patients whose tumor does not achieve at least partial response during induction chemotherapy, andpatients with residual cancer disease within fifth week after the end of radiotherapy will be evaluated for surgical resection of the tumor.
Likewise, in patients with partial or complete response, radical or modified radical emptying will be performed in all cases of N2 (a, b or c) or N3 at diagnosis. Extended supraomohyoid emptying for level IV will be performed in cases N0 and N1 submitted to rescue surgery after failure of neoadjuvant chemotherapy or raditorerapy at 5040 cGy.

The surgical technique used basically consists of:

- Cervical retail including platysma. Extension to supraclavicular fossa in cases of radical emptying.

- Contralateral cervical emptying when indicated.

- Homolateral cervical emptying when indicated.

- Resection of the primary tumor when indicated (rescue).

- Reconstruction with myocutaneous flap or microsurgical transplantation, when more than 50% of the mucosa of the pharynx is resected.

**Evaluation at the end of treatment**

1. An head and neck CT scan and / or MRI will be performed 6 - 12 weeks after the end of radiotherapy.

2.It will be offered the option of performing PET-CT 8 to 12 weeks after radiotherapy ends, when reassessment by the investigator of the Department of Head and Neck judge it as helpful for tumor response evaluation. The head and neck Surgeon will discuss aspects related to the surgical approach of the neck and primary tumor.

 Follow-up visits of patients in the post-treatment period

a) Monthly in the first 6 months;

b) Bimonthly for an additional 6 months;

c) Quarterly for an additional 2 years;

d) Semiannual for 2 additional years.

Annual imaging (computed tomography or cervical magnetic resonance imaging and chest X-ray) or as indicated.

**Discontinuations**

Reasons for discontinuation should be documented.

**Pathologic anatomy**

Pretreatment:

- HE stained biopsy material with histological gradation.

- Matarial biopsy kept in a tumor bank

After treatment

- Biopsies and material from rescue surgeries stained by HE.

Examination of operative parts

- All pieces of resection of the primary tumor will be studied in serial cuts

- All dissected lymph nodes will be classified according to the level, number, diameter and presence of capsule rupture

- In the primary tumor will be studied histologicaly, describing the degree of differentiation, presence of necrosis, perineural infiltration and vascular embolization.

Measures of effectiveness

Measurability of disease

Two-dimensional measurable lesions

a- Physical examination: nodule or superficial lymph node with at least 20X10 mm.

b- Computed tomography: lesion of at least 10 X 10 mm

One-dimensional measurable lesions

They include all lesions where only one diameter can be measured

a- Physical examination:> 20 mm

b- Computed tomography:> 20 mm

Measurable non-measurable lesions

Non-uni or two-dimensional measurable lesions as defined above (e.x .: primary tumors).

 Criteria for evaluation of response

As for the tumor response

All patients receiving> 2 cycles of induction chemotherapy, and in those with a response, a second evaluation will be on the fifth week of radiotherapy.

Response criteria - WHO and modified WHO

All uni or two-dimensional lesions measurable by physical examination should be measured after the second cycle of chemotherapy, as mentioned above.

In case of suspected progression before the end of induction chemotherapy, or in a subsequent moment during treatment with radiotherapy (up to 5040 cGy), oroscopy, nasopharyngoscopy and / or computed tomography or MRI as clinically indicated should be repeated. However, in order to define the overall response of the patient, the measurement considered will be that done after the end of the treatment.

Uni or two-dimensionally measurable disease

Complete response (CR): disappearance of any evidence of detectable cancer disease, either clinically, radiologically or by endoscopy / biopsies.

Partial response (RP): in the case of a two-dimensionally measurable disease, reduction of at least 25% or 50% of the sum of the products of the larger perpendicular diameters in all measurable lesions, in accordance with modified WHO and WHO criteria, respectively. Patients whose tumors achieve partial response under any criteria will be selected for following the third induction chemotherapy followed by radiotherapy. In case of uni-dimensionally measurable lesions, reduction by at least 30% of the sum of the diameters of all the lesions. It is not necessary that all injuries regress to qualify RP, but no injury may have progressed or appeared.

Stable disease (SD): for two-dimensional measurable disease <50% decrease and <25% increase in summation of the products of the largest perpendicular diameters of all measurable lesions observed prior to treatment. For unidimensionally measurable disease, <50% reduction and <25% increase in the sum of the diameter of all lesions observed prior to treatment. No new lesions should appear.

Progressive disease (PD):> 25% increase in size of at least one measurable lesion compared to baseline measurements, or appearance of a new lesion.

Unmeasurable disease

Complete response: complete disappearance of all lesions.

Partial response: estimated reduction of tumor size by 50%.

Stable disease: estimated decrease of less than 50% or estimated increase less than 25%.

Progressive disease: appearance of any new lesions not previously identified or estimated increase greater than 25% in existing lesions.

Pathological response

| **Reponse**  **(bi-dimensionally measurable lesions)** | **Response (unidimensional measurable lesions)** | **Response**  **(non-mensurable lesions)** | **global**  **response** |
| --- | --- | --- | --- |
| Progressive disease | Any | any | Progressive disease |
| any | Progressive disease | any | Progressive disease |
| Stable  Disease | Any, except progressive disease | Any, except progressive disease | Stable  disease |
| Partial response | Any, except progressive disease | Any, except progressive disease | Partial  response |
| Complete Response | Complete Response | Complete  Response | Complete Response |
|  |  |  |  |
| Any | Any | Progressive disease or a new lesion | Progressive disease |
| absence | Partial response | Any, except progressive disease | Partial  response |
| Absence | Complete response | Any, except progressive disease and stable disease | Partial response |
| Absence | Complete response | Complete response | Complete response |

Complete response: complete response documented in biopsies or surgical specimen.

Partial response: presence of disease in the surgical specimen or in biopsies, in patients with complete or partial response verified before surgery.

Determining the overall response:

The duration of the best response will be from the date of initiation of treatment to documentation of disease progression.

Time to disease progression

From the date of initiation of treatment to progression, death, last contact or last evaluation of the tumor prior to initiation of subsequent antitumor treatment (rescue).

Adverse events

Any undesirable event associated with the use of a drug, whether or not it is related to the drug, and includes any side effects, toxicity or sensitivity reactions. Includes any clinical or laboratory event not normally seen in the patient.

They will be graded and graded with NCI's expanded common toxicity criteria (Appendix).

Dose corrections of the citotoxic chemotherapy agents as well as discontinuation of study patients will be at the discretion of the investigators. The taking of any of the above actions should be documented in the patient's medical register.

Definitions:

Preservation rate (to be evaluated in patients considered to be candidates for surgery with curative intent at diagnosis): obtaining local disease control without the need for surgical intervention on the primary site of the disease. It will be evaluated 4 weeks after the end of radiotherapy (7040 Gy).

Prohibitive toxicity: a lethal event or serious adverse event that results in the need for hospitalization for treatment.

Statistical analysis

Simple ratios will be used to describe the distribution of categorical variables. The distribution of numerical variables will be described by median and range. Survival curves will be generated for time-to-event variables according to the Kaplan-Meier method and compared among different group using the log-rank method. Bootstrap analysis will be used to perform internal validation of the findings. The maximum standardized log-rank statistic will be applied in order to optimize the maximum SUV decrease cut-off value to consider a patient as having a responsive tumor. Univariate Cox’s proportional hazard models will be generated in order to calculate the Hazard ratios (and corresponding confidence intervals). Statistical analysis will be performed using the softwares SPSS and Project R.

References

1. Jemal A, Siegel R, Ward E, et al. Cancer statistics. CA Cancer J Clin 2007;57:43-66.

2. Seiwert TY, Cohen EE. State-of-the-art management of locally advanced head and neck cancer. Br J Cancer 2005;92:1341-8.

3. Kowalski LP, Franco EL, Torloni H, Fava AS, et al.Lateness of diagnosis of oral and oropharyngeal carcinoma: factors related to the tumour, the patient and health professionals. Eur J Cancer B Oral Oncol. 1994;30B:167-73.

4.Carole Fakhry et al.Clinical Implications of Human Papillomavirus in Head and Neck Cancers. J Clin Oncol 2006; 24: 2606.

5. Jeremic B, et al: Hyperfractionated radiation therapy with or without concurrent low-dose daily cisplatin in locally advanced squamous cell carcinoma of head and neck: A prospective randomized trial. J Clin Oncol 2000; 18(7): 1458-1464.

6. Wendt TG, et al: Simultaneous Radiochemotherapy versus Radiotherapy Alone in Advanced Head and Neck Cancer: A Randomized Multicenter Study. J Clin Oncol 1998, 16: 1318-1324.

7. Pignon JP, et al: Chemotherapy added to locoregional treatment for head and neck squamous-cell carcinoma: Three meta-analyses of updated individual data. Lancet 2000; 355 (9208): 949-955.

8. The Department of Veterans Affairs Laryngeal Cancer Study Group. Induction chemotherapy plus radiation compared with surgery plus radiation in patients with advanced laryngeal cancer. N Engl J Med 1991; 324:1685-90

9. Forastiere AA, et al: Concurrent Chemotherapy and Radiotherapy for Organ Preservation in Advanced Laryngeal Cancer. N Engl J Med 349 (22): 2091-8, 2003.

10. Lefebvre JL, et al: Larynx preservation in pyriform sinus cancer: Preliminary results of a European Organization for Research and Treatment of Cancer phase III Trial. EORTC Head and Neck Cancer Cooperative Group. Natl Cancer Inst 1996;88 (13):890-899.

11. Domenge C, et al: Randomized trial of neoadjuvant chemotherapy in oropharyngeal carcinoma. French Groupe d’Estude des Tumeurs de la Tete et du Cou (GETTEC). Br J Cancer. 83 (12) : 1594-1598, 2000.

12. Calais G, et al: Final Results of the 94–01 French Head and Neck Oncology and Radiotherapy Group Randomized Trial Comparing Radiotherapy Alone With Concomitant Radiochemotherapy in Advanced-Stage Oropharynx Carcinoma. J Clin Oncol 2004; 22(1):69-76.

13. Hitt R, López-Pousa A, Martínez-Trufero J, et al. Phase III Study Comparing Cisplatin Plus Fluorouracil to Paclitaxel, Cisplatin, and Fluorouracil Induction Chemotherapy Followed by Chemoradiotherapy in Locally Advanced Head and Neck Cancer. J Clin Oncol 2005; 23:8636-8645.

14. Schrijvers D, et al: Docetaxel, cisplatin and 5-fluorouracil in patients with locally advanced unresectable head and neck cancer: a phase I-II feasibility study. Ann Oncol 2004; 15:638-645.

15. Vermorken JB, Remenar E, van Herpen C, et al. Cisplatin, Fluorouracil, and Docetaxel in Unresectable Head and Neck Cancer. N Engl J Med 2007; 357:1695-704.

16. Posner MR, Hershock DM, Blajman CR, et al. Cisplatin and Fluorouracil Alone or with Docetaxel in Head and Neck Cancer. N Engl J Med 2007; 357:1705-15.

17. Hitt R, Grau JJ, Lopez-Pausa A, et al. Randomized phase II/III clinical trial of induction chemotherapy (ICT) with either cisplatin/5-fluorouracil (PF) or docetaxel/cisplatin/5-fluorouracil (TPF) followed by chemoradiotherapy (CRT) vs. crt alone for patients (pts) with unresectable locally advanced head and neck cancer. Journal of Clinical Oncology, 2006 ASCO Annual Meeting Proceedings Part I. Vol 24, No. 18S (June 20 Supplement), 2006: 5515.

18. Lorch JH, Posner MR, Wirth LJ, et al. Induction Chemotherapy in Locally Advanced Head and Neck Cancer: A New Standard of Care? Hematol Oncol Clin N Am 2008; 22:1155-1163.

19. Therasse P, Arbuck SG, Eisenhauer EA, et al. New Guidelines to Evaluate the Response to Treatment in Solid Tumors. JNCI 2000; 92(3): 205-16.

20. Miller AB, Hoogstraten B, Staquet M, et al. Reporting results of cancer treatment. Cancer 1981; 47(1):207-14.

21. Burton C, Ell P, Linch D. The role of PET imaging in lymphoma. Br J Haematol. 2004 Sep;126(6):772-84. Review.

22. Beyer T, Townsend DW, Blodgett TM. Dual-modality PET/CT tomography for clinical oncology. Q J Nucl Med. 2002 Mar;46(1):24-34.

23. von Schulthess GK, Steinert HC, Hany TF. Integrated PET/CT: current applications and future directions. Radiology. 2006 Feb;238(2):405-22.

24. Kostakoglu L, Goldsmith SJ, Leonard JP et al. FDG-PET after 1 cycle of therapy predicts outcome in diffuse large cell lymphoma and classic Hodgkin disease.Cancer. 2006 Dec 1;107(11):2678-87.

**Free and Informed Consent Term (TCLE)**

**Hospital A.C.Camargo- Antônio Prudente Foundation**

**Rua Professor Antonio Prudente, 211 Telephone: (011) 2189-5000**

**SãoPaulo-SP CEP: 01509-010**

**POST-INFORMED CONSENT**

**(Mandatory for Clinical Research in Humans - Resolution 196/96 and CNS Resolution 252/97 of the Ministry of Health)**

**This term is being requested exclusively for participation in this research, without possibility of extension of the same authorization for other projects.**

**DESIGN: PET-SCAN USE AS A PREDICTOR OF EFFECTIVENESS FOR LOCOREGIONAL CONTROL IN CARCINOMA EPIDERMÓIDE OF HEAD AND NECK.**

**1. Identification data of the patient or legal guardian**

**Patient's Name: ____________________________________________**

**Gender: male () female () Date of birth _ / _ / _**

**Identity document No: __________________________**

**Address:_______________________________________________**

**City State:___________**

**CEP: _________________ Tel.:________________________**

**Responsible: ______________________________________________**

**Gender: male () female () Date of birth _ / _ / _**

**Identity document No: __________________________**

**Address:_________________________________________________**

**City State:___________**

**CEP: _________________ Tel.:________________________**

**2. Objectives of the study**

**You have been invited to participate in this study in head and neck cancer. Treatment of locally advanced head and neck cancer is based on chemotherapy with cisplatin and 5-fluoro-uracil concomitant with taxanes followed by radiotherapy with or without chemotherapy. The Clinical Staff of the Clinical Oncology Department of AC Camargo Hospital is developing clinical research in the area of ​​locally advanced head and neck cancer submitted to standard chemotherapy treatment (cisplatin and 5-fluorouracil associated with taxane) followed by concurrent or non-radiotherapy chemotherapy. The aim is to identify early response factors to treatment and to correlate relapse-free survival with functional imaging tests (PET-CT) and to compare with computed tomography, a traditional imaging test to evaluate response to treatment.**

**3. Description of procedure and duration of study participation**

**The treatment scheme proposed in this study, as well as the conventional laboratory and imaging examinations proposed, is routine in the Department of Clinical Oncology, and no experiment is considered. Additionally, PET-CT will be requested, which is an imaging exam that associates glucose metabolism images with anatomical images of computed tomography without contrast, pre-therapy, after the 1st cycle of chemotherapy and after the end of radiotherapy. There will be 3 or 4 cycles of chemotherapy every 21 days each. You will leave the study if you develop disease progression or toxicity that prevents further therapy. PET-CT is expiremental in this study, but it is not expiremental in oncology, already part of the diagnostic oncology propaedeutics, performed in the Department of Imaging / Nuclear Medicine of the same institution and with low risk of complications inherent to the examination. On the day of the test, you will receive intravenous administration of a specific, pre-ordered material called 18F-FDG and produced by IPEN-CNEN-SP. This material is regularly used in the Nuclear Medicine sector to perform this examination without any complications and will be donated by IPEN for the examination. Before the venous injection of 18F-FDG you will have your blood glucose checked by performing a hemoglicotete test that uses digital capillary puncture to make sure that blood glucose levels are below 200 mg / dl, because above these the quality of the exam may be impaired.**

**You should have at least 3 hours of your day to perform the previously marked examination, as you should remain for about 30 minutes at rest before the injection of 18F-FDG, wait about 90 minutes to start the examination after the injection of 18F -FDG and about 50 minutes to 1 hour to obtain the images of the examination and verification of their technical quality. I emphasize, however, that this period may be extended if there is a delay in the delivery time of the material to the institution and there is a routine orientation in scheduling this examination in the sector to patients who perform this type of examination so that they do not undertake appointments with scheduled schedules on this day.**

**Potential risks**

**Risks inherent in the usual treatment, even if not participating in this protocol:**

**Most frequent side effects from the use of chemotherapy:**

**- Docetaxel: nausea, vomiting, mucositis, falling white blood cells and infection, muscle pain and hair loss.**

**- 5 Fluorouracil: mucositis, diarrhea, palmoplantar syndrome, pancytopenia, myocardial ischemia, mild grade hair loss**

**- Cisplatin: nausea, vomiting, renal toxicity, auditory toxicity, neuropathy, ischemia, falling red blood cells, falling white blood cells or platelets.**

**- Carboplatin: nausea, vomiting, renal toxicity, auditory toxicity, falling red blood cells, falling white blood cells or platelets.**

**Side Effects Related to Radiotherapy:**

**- mucositis, odontogenic infection, dermatitis or bone necrosis.**

**Effects related to computed tomography:**

**- allergic reaction to contrast, phlebitis.**

**Regarding PET-CT, which will be the only additional procedure to standard therapy and examinations that the patient would normally receive out of the study, the predicted risks are those involved with the pain sensation that may occur when the digital needle puncture is performed with needle of insulin to check glycemia and venous puncture with scalp for 18F-FDG injection. There is also the risk of venous extravasation of the venous injected material but, if it does occur, it will not involve any organ lesion and also the risk of possible infection after the mentioned punctures, but which are minimized with adequate asepsis.**

**Female patients of childbearing potential should ensure that they will not become pregnant during therapy because of the teratogenic potential of drugs and radiation (imaging), therefore assuming the risks of gestation, should they occur.**

**Expected benefits**

**Participation in this study is intended to improve the treatment approach of patients with locally advanced head and neck cancer. If you agree to participate in the study there may or may not be a direct benefit for you. We hope that the information obtained in this study with the inclusion of the imaging method (PET-CT) early in the evaluation of these patients will allow us to identify patients who will actually benefit from the chemotherapy treatment followed by radiotherapy and, in future studies, this can be confirmed and avoid toxicity of these drugs in patients who do not get the appropriate response expected with the initial treatment instituted.**

**Alternative treatments to the research object**

**When we opt for chemotherapeutic treatment followed by radiotherapy associated or not with chemotherapy, the schedules and doses will be the traditional ones used in our institution. The treatment offered is not under test.**

**Safeguarding confidentiality, confidentiality and privacy**

**The eventual inclusion of the results in scientific publication will be done in such a way as to maintain their anonymity. You will have access to your exam data, medical care and therapy administration when requested.**

**Clarifications on compensation or damages related to research**

**You will not have any type of remuneration when you agree to participate in this study. The survey does not involve any form of financial compensation to participants. There is no compensation for complications caused by the treatment.**

**Clarifications on other rights of the subject subject to the research**

**Your participation in the study is voluntary. You have the right to leave the study at any time for any reason. If you leave the study or decide not to participate in the study, your treatment will not be impaired. However, if you decide to leave the survey, you should inform your doctor.**

**The Medical Ethics Committee of the A.C.Camargo Hospital is the legal responsible for certifying that patients' rights are protected. This committee reviewed and approved this study.**

**Information about names, phones and addresses for contacts**

**Clarifications to questions about the rights of research participants and / or damage related to the research, contact the researchers Dr Ulisses Ribaldo Nicolau (011) 99011147 or 21895000 r 1551 or Dr Tadeu Ferreira de Paiva Júnior (011) 84913815 or 21895000 r 1551. If the principal investigator does not provide sufficient information / clarification, please contact the coordinator of the ACCamargo-SP Hospital Ethics Committee at 2189-5020.**

**You will receive a copy of this document and the original will be filed in the doctor's chart. Only sign this document if you fully consent to its terms.**

**Free and Informed Consent**

**I declare that I have read and understood the procedure. I further declare that I have discussed this procedure with my physician. I understand the purpose of the study and the methods that will be used. I also understand that my entry into this study is voluntary.**

**Signature of patient or legal guardian ___________________________**

**Date: ____ / ____ / ____**

**Patient's name ___________________________________ RG ___________**

**MEDICAL DECLARATION OBTAINING POST-INFORMED CONSENT**

**I declare that I have explained this procedure, with all the necessary details for the patient (or legal guardian) ___________________________________.**

**In my judgment, there was access to all available information, including the risks and benefits so that an informed decision could be made.**

**Signature of Physician __________________________ Date: _____ / ____ / ____**
