# Supplementary material for: Early metabolic 18F-FDG PET/CT response of locally advanced squamous-cell carcinoma of head and neck to induction chemotherapy: A prospective pilot study
Source: PLoS One. 2018 Aug 16;13(8):e0200823. doi: 10.1371/journal.pone.0200823 (PMC6095513; doi:10.1371/journal.pone.0200823)
Supplement: S2 File — (DOCX) [file pone.0200823.s004.docx]

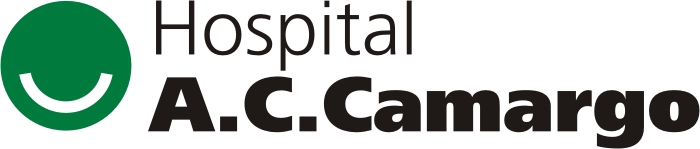


**USO DO PET-SCAN COMO PREDITOR DE EFICÁCIA PARA CONTROLE LOCOREGIONAL E SOBREVIDA EM CARCINOMA EPIDERMÓIDE DE CABEÇA E PESCOÇO**

**Pesquisador Responsável:**

Ulisses Ribaldo Nicolau

**Co-pesquisadores:**

Tadeu Ferreira de Paiva Jr

Paula Nicole Vieira Pinto

Antonio Cássio de Assis Pellizzon

Marcello Ferretti Fanelli

Eduardo Nóbrega Pereira Lima

Luiz Paulo Kowalski

São Paulo, julho de 2009

**USO DO PET-SCAN COMO PREDITOR DE EFICÁCIA PARA CONTROLE LOCOREGIONAL E SOBREVIDA EM CARCINOMA EPIDERMÓIDE DE CABEÇA E PESCOÇO.**

**Introdução**

O carcinoma espino celular (CEC) de cabeça e pescoço representa 5% das neoplasias recém diagnosticadas, 644.000 casos e 350.000 mortes por ano no mundo^1^. Nessa localização anatômica, os tumores ocorrem mais frequentemente em cavidade oral (44%), laringe (31%) e faringe (25%). A maioria dos casos apresenta-se em tumores localmente avançados potencialmente curáveis. Metástases à distância ao diagnóstico ocorrem em até cerca de 10 % dos casos. À despeito dos avanços no tratamento, a sobrevida livre de doença (SLD) e sobrevida global (SG) permanecem insatisfatórias. Cerca de 40% a 60% e 20% a 30% dos pacientes terão recaída loco-regional e sistêmica respectivamente. Dentre os fatores implicados no prognóstico destes pacientes, destacam-se o estadiamento, condição clínica do paciente e fatores tais como tabagismo, uso de álcool e infecção pelo HPV em tumores de orofaringe, sendo este último grupo aparentemente portador de melhor prognóstico quando comparado a pacientes tabagistas e etilistas^2, 3,4^.

O tratamento dos tumores de cabeça e pescoço é altamente complexo e varia de acordo com diferentes sítios anatômicos, a dificuldade de acesso a esses sítios e da importância em preservar órgãos e estruturas. Em geral, pacientes com tumores iniciais são tratados por cirurgia ou radioterapia, enquanto aqueles com tumores avançados recebem terapias combinadas, combinando-se em variadas sequências cirurgia radioterapia e quimioterapia. Nos anos recentes, quimioterapia tem assumido um papel maior no tratamento da doença localmente avançada, sendo utilizada em diferentes estratégias: indução ou neoadjuvante, concomitante à radioterapia e adjuvante, isoladas ou em combinação. A preservação de órgãos com tratamentos clínicos pela associação de quimioterapia e radioterapia, reservando-se ressecção cirúrgica aos não respondedores é uma realidade, entretanto, aguardam-se resultados consolidados da seqüência ideal dessas modalidades, em termos de controle local e sobrevida. Seleção rigorosa de pacientes, monitorização de efeitos colaterais e métodos para avaliar pacientes responsivos ao tratamento são importantes no intuito de minimizar a toxicidade. Tomografia com emissão de pósitrons (PET-CT) constitui método de auxílio diagnóstico, estadiamento e avaliação de resposta para neoplasias em crescente uso na prática clínica. Dentre os isótopos em uso e em investigação, vem se ampliando o emprego radiotraçador 18-F-fluorodeoxiglicose (18-F-FDG). Consideradas as possibilidades de benefício do uso do PET-CT em carcinoma epidermóide de cabeça e pescoço, destacam-se seu auxílio no planejamento de radioterapia, além de posteriores tomadas de decisões terapêutica com relação a esvaziamento cervical e abordagem do tumor primário ao término do tratamento irradiante, questões estas que ganham relevância pelo crescente uso de radioterapia associada à quimioterapia nesta entidade.

Observa-se ainda o potencial uso deste método para avaliação precoce de terapias instituídas. Ao se predizer precocemente à eficácia de terapias de elevada toxicidade ao início das mesmas, busca-se o benefício de selecionar uma fração de pacientes que possam ser poupados de tratamentos que acarretarão morbidade, com posssibilidade de alternar-se precocemente para terapias alternativas.

**Histórico**

**1- Associação Radioterapia e quimioterapia em Carcinoma Espinocelular de Cabeça e Pescoço**

Durante a década de 90 diversos estudos avaliaram a associação de quimioterapia concomitante a radioterapia, demonstrando maior eficácia no controle locoregional e conseqüente possível impacto em sobrevida livre de recorrência e sobrevida global^5,6,7^.

Jeremic e colaboradores randomizou 130 pacientes com diagnóstico de CEC de cabeça e pescoço localmente avançado para estudo fase III contendo 02 braços, sendo o braço controle composto por pacientes submetidos a radioterapia hiperfracionada, 77 Gy, contra pacientes submetidos ao mesmo regime radioterápico associado a cisplatina diária, 6mg/m2 durante a radioterapia. Sobrevida global de 68% versus 49% em 2 anos, e 46% versus 25% em 5 anos (p=0,0075) foi alcançado para o braço que realizou tratamento combinado em relação a radioterapia exclusiva. Significativos ganhos em sobrevida livre de progressão, sobrevida livre de progressão loco regional e supressão de metástase à distância também foram observados no grupo que recebeu tratamento combinado concomitante^5^.

Corroborando com estudos adicionais que demonstraram vantagem para controle local e sobrevida para a modalidade de quimioterapia concomitante a radioterapia, metanálise publicada por Pignon e colaboradores em 2000 evidenciou benefício na associação de quimioterapia ao tratamento local para CEC localmente avançado em cabeça em pescoço.

Avaliados 63 estudos contendo 10 741 pacientes, submetidos a tratamento locoregional com ou sem quimioterapia, observou-se ganho significativo de sobrevida de 4% em 2 e 5 anos em favor da associação de quimioterapia^7^.

**Laringe**

Em 1991, foi publicado importante estudo em que se avaliou o uso de quimioterapia de indução, composto por cisplatina associada à infusão contínua de 5-fluorouracil, seguida de radioterapia nos pacientes responsivos; tendo-se no braço controle o tratamento então standard, consistindo em laringectomia total seguida de radioterapia adjuvante.

Dentre os importantes achados, observou-se uma taxa de 64% de preservação de laringe, sem decréscimo em sobrevida no grupo de pacientes tratados com quimioterapia de indução seguido de radioterapia quando comparado ao braço controle (sobrevida em 2 anos de 68% em ambos os grupos). Observou-se ainda 85% dos pacientes responsivos após 02 ciclos de quimioterapia, sendo 31% de resposta total^8^.

No cenário de preservação de órgão, Forastiere e colaboradores avaliaram num estudo de 3 braços, quimioterapia de indução versus a associação concomitante de cisplatina e radioterapia versus radioterapia exclusiva para pacientes com diagnóstico de CEC de laringe localmente avançado candidatos a laringectomia total com intuito curativo. A despeito de sobrevida global semelhante nos três grupos, a associação concomitante de quimioterapia e radioterapia mostrou-se superior a quimioterapia de indução ou radioterapia exclusiva para controle locoregional e preservação de laringe, tendo-se observado, entretanto tendência de menor taxa de recorrência à distância para os 02 grupos que receberam quimioterapia com relação ao braço que recebeu radioterapia isolada^9^.

**Hipofaringe**

Lefebvre e colaboradores avaliaram o uso de quimioterapia de indução seguida de radioterapia em pacientes responsivos, nos mesmos moldes de estudo realizado pelo “Veterans Affairs Latyngeal Cancer Study Group”, para pacientes portadores de CEC localmente avançado de hipofaringe, passíveis de ressecção cirúrgica por faringolaringectomia total, com dissecção cervical radical, randomizados entre quimioterapia de indução seguida de radioterapia (reservada cirurgia seguida de radioterapia para pacientes não responsivos) ou cirurgia seguida de radioterapia (braço controle).

O grupo submetido à quimioterapia de indução obteve taxa de resposta completa de 54% no tumor primário e 51% para linfonodos cervicais, além de taxa de preservação de laringe em 3 e 5 anos de 42% e 35% respectivamente, sem que se observassem diferenças significativas em recorrência loco-regional quando comparado ao grupo cirúrgico. Notou-se ainda tendência de menor taxa de recorrência a distância no grupo submetido à quimioterapia de indução, sem diferença significativa na sobrevida global^10^.

**Orofaringe**

Domenge e colaboradores avaliou o papel da quimioterapia de indução com uso de cisplatina e 5-fluorouracil seguido de abordagem locoregional consistindo de cirurgia seguida de radioterapia ou radioterapia exclusiva, versus braço controle composto por tratamento consistindo em cirurgia seguida de radioterapia, para pacientes com diagnóstico de CEC localmente avançado de orofaringe passíveis de tratamento curativo por cirurgia ou radioterapia. Após 318 pacientes avaliados, observou-se ganho significativo na sobrevida mediana do grupo tratado com quimioterapia de indução quando comparado ao braço que não recebeu quimioterapia, com sobrevida mediana de 5,1 anos e 3,3 anos (p= 0.03) respectivamente^11^.

Calais e colaboradores avaliou a adição de carboplatina e 5-fluorouracil infusional concomitante a radioterapia sobre braço controle que recebeu radioterapia exclusiva em pacientes com CEC de orofaringe estádios clínicos III e IV.

Avaliados 266 pacientes, o grupo tratado pelo regime combinado concomitante alcançou ganho em SG 5 anos, em relação ao grupo controle, radioterapia exclusiva ( 22% versus 16%, p=0,05, respectivamente). Sobrevida doença-específica (27% versus 15%, p=0,01) e controle loco-regional (48% versus 25% p=0,002) também favoreceram o grupo que recebeu tratamento combinado^12^.

**2- Quimioterapia de indução**

Na década de 90, quimioterapia de indução, seguida de radioterapia estabeleceu-se como tratamento opcional factível para pacientes com CEC de cabeça e pescoço localmente avançado candidatos a preservação de órgão. Apesar dos potenciais benefícios dessa estratégia, incluindo potencial para supressão de metástase a distância, tal associação falhava em alcançar benefício em sobrevida global quando comparado a radioterapia exclusiva ou radioterapia concomitante à quimioterapia.

Frente ao consolidado uso concomitante da radioterapia à quimioterapia nas duas últimas décadas, a quimioterapia de indução voltou a ser discutida pela emergência de novos agentes tais como taxanes, com interesse crescente na associação de tais quimioterápicos a regimes anteriormente utilizados, visando explorar seus potenciais benefício terapêuticos, tais quais maiores taxas de resposta, supressão de metástases a distância e potencial impacto em sobrevida.

Dentre os diversos agentes, ganha destaque a associação de docetaxel ou paclitaxel à cisplatina e 5-fluorouracil.

Hitt et al, em 2005, estudou 382 pacientes com CEC de cabeça e pescoço ressecável ou irressecável. Foi comparado Cisplatina 100mg/m² D1 e 5FU 1000mg/m² D1 a D5 versus Cisplatina 100mg/m² D2, 5FU 500mg/m² D2 ao D6 e Paclitaxel 175mg/m² D1, aplicados a cada 21 dias, por 3 ciclos. Em ambos os braços, pacientes com resposta completa ou resposta parcial maior que 80%, a quimioterapia de indução foi seguida por radioterapia 70Gy concomitante à Cisplatina 100mg/m² D1, D22 e D43.

O grupo que recebeu Paclitaxel apresentou maior taxa de resposta completa (33% vs 14%, p<0.001), além de tendência a melhor SG (43 meses vs 37 meses, p=0,06)^13^.

Estudo fase I/II publicado em 2004 avaliou o uso de docetaxel, cisplatina e 5-fluorouracil em dois níveis de dose. Grupo avaliado para maior dose recebeu cisplatina 100mg/m2 e docetaxel 75mg/m² no d1, associados a 5-Fluorouracil 750mg/m2/24h em infusão contínua por 96 horas, contra regime com idênticas doses de 5-Fluorouracil e docetaxel, porém com uso de cisplatina 75mg/m² no d1. Houve melhor perfil de toxicidade no esquema que utilizou cisplatina 75mg/m², além de menor incidência de neutropenia febril após a inclusão de ciprofloxacina profilático^14^.

Considerando o estudo prévio, tal regime, docetaxel, cisplatina e 5-fluoracil, (TPF) foi avaliado por Vermorken e colaboradores que o comparou à associação cisplatina-5-fluorouracil (PF) previamente (neoadjuvante) à radioterapia em CEC de cabeça e pescoço localmente avançado considerado irressecável. Reservou-se abordagem cirúrgica ao tumor primário se observada doença residual, assim como esvaziamento cervical para lesões cervicais N2-3 à despeito de resposta terapêutica, ou para doença residual.

Avaliados 358 pacientes, 177 em braço que recebeu TPF e 181 para o braço que recebeu PF por 4 ciclos previamente a radioterapia, o braço com 03 drogas apresentou ganhos em sobrevida livre de progressão tumoral (11,0 meses versus 8,2 meses, p=.007), traduzindo-se ainda em ganho significativo de sobrevida global para o braço contendo 03 drogas (SV mediana: 18,8 meses VS 14,5 meses, p=0,02)^15^.

Em estudo prospectivo randomizado, publicado por Posner et al, demonstrou-se que quimioterapia de indução utilizando TPF, quando comparado a PF, seguido de radioterapia associado a carboplatina semanal radiosensibilizante, foi mais eficiente em termos de SLD 3a (49% VS 37%, p=0,004) e SG 3a (62% vs 48%, p=0,006). A maioria dos pacientes era portadora de CEC localmente avançado de orofaringe (53%), seguidos dos sítios laringe (17%), cavidade oral (15%) e hipofaringe (14%). Trinta e cinco porcento foram considerados irressecáveis e 65% ressecáveis (31% considerados de baixa curabilidade e 34% com intuito de preservação de órgão)^16^.

Em um elegante estudo, 295 pacientes (amostra planejada de 386 pacientes) com CEC localmente avançado, irressecáveis, de cabeça e pescoço foram alocados num estudo randomizado, o qual foi apresentado por Hitt et al na ASCO 2006, onde foi comparado TPF de indução versus PF, seguido em ambos os braços por radioterapia concomitante à cisplatina. O estudo incluiu um terceiro braço, que consistia na associação concomitante exclusiva de cisplatina e radioterapia. Pacientes que receberam quimioterapia de indução alcançaram resposta completa de 70% contra 49% para pacientes que foram randomizados para quimioradioterapia concomitante exclusiva, levando a um ganho em sobrevida livre de progressão para quimioterapia de indução, mais importante para o regime TPF (16 meses), contra PF (12 meses) seguido de quimioradioterapia, em detrimento de 8 meses para quimioradioterapia exclusiva (p= 0.002)^17^.

O estudo publicado por Hitt e colaboradores^17^ portanto discute a questão de possível substituição da política de quimioterapia concomitante a radioterapia pelo uso de quimioterapia de indução seguido de quimioterapia e radioterapia concomitantes, já que inclui em 1 dos 03 braços o grupo controle com terapia concomitante, sem uso de quimioterapia de indução.

Agregados os dados acima mencionados, observa-se a tendência em muitos centros de uso de docetaxel com cisplatina e 5-fluorouracil como regime de indução, política esta embasada ainda pelos estudos denominados TAX 323 e TAX 324^18^.

**Avaliação de resposta em carcinoma epidermóide de cabeça e pescoço**

Em conformidade com outros tumores sólidos, carcinoma epidermóide de cabeça e pescoço tem avaliada resposta tumoral à quimioterapia e radioterapia por critérios tais quais RECIST^19^, com mensuração unidimensional de lesões e comparações pós terapêuticas, ou por critério da Organização Mundial da Saúde (WHO) ^20^ e suas variantes^16^. Assim, estudo recente conduzido por Posner e colaboradores^16^, utilizou o critério WHO modificado, cuja peculiaridade consistia em considerar resposta parcial (RP) decréscimo de 25% ou mais do produto dos maiores diâmetros das lesões mensuráveis^16^. Consideravam-se portanto achados de imagem (tomografia computadorizada ou ressonância nuclear magnética) para o tumor primário e disseminação cervical, assim como achados por propedêutica armada (p.ex nasofibrolaringoscopia). Vale observar o critério mais frequentemente utilizados nestes estudos, que considera resposta parcial redução de pelo menos 50% no produto das lesões mensuráveis^20^. Caracteriza-se assim, ao segundo ciclo da quimioterapia de indução, aqueles que sendo quimiosensíveis se submeteriam ao terceiro ciclo de quimioterapia e posterior associação de radio e quimioterapia concomitantes. Observa-se ainda que o critério de quimiosensibilidade ao segundo ciclo e a suposição de radiosensibilidade por este critério constituiu e orientou a maior parte dos estudos de quimioterapia de indução nas duas últimas décadas. Assim, a associação de radioterapia à quimioterapia seguida de discussão da abordagem cirúrgica posterior para o tumor primário e pescoço dependem do estadiamento pelo TNM e avaliação subseqüente pela resposta tumoral de acordo com os citados critérios WHO ou RECIST, que se baseiam em achados de imagem e se complementam com avaliações clínico laboratoriais. Apesar do papel estabelecido nesta associação terapêutica para pacientes nos estádios III e IV, elevadas taxas de recidiva ou de baixa resposta tumoral ao tratamento adicional focam a necessidade de avaliação precoce de pacientes responsivos. A seleção ao início do tratamento de pacientes com baixa probabilidade de resposta ao tratamento quimioradioterápico possibilita que os mesmo tenham discutida sua estratégia terapêutica visando reavaliação precoce de ressecabilidade, com o benefício de não expô-los a tratamento de elevada toxicidade e baixa probabilidade de sucesso. Neste cenário, exames não invasivos e que associem característica funcionais tem o potencial benefício de avaliar precocemente a eficácia de terapias em curso. As principais falhas consideradas do PET na avaliação dos tumores são a ausência de marcadores anatômicos e a dificuldade de diferenciar atividade inflamatória, que também incorpora o FDG, de atividade tumoral^21^

Entretanto, recentemente, o protótipo do PET acoplado à CT foi introduzido permitindo a obtenção de imagens co-registradas do PET e CT numa única sessão de exame^22^. A combinação destas modalidades de exame melhorou a identificação e definição das anormalidades identificadas pelo FDG-PET e a associação de informações biológicas com as anatômicas ofereceu melhor definição das anormalidades teciduais^23^.

Análises retrospectivas e estudos prospectivos tentam estabelecer o papel do FDG-PET no estadiamento de carcinoma epidermóide de cabeça e pescoço. Em centros que dispõem desta técnica, cresce a sua utilização no seguimento destes pacientes após o tratamento do tumor primário, usualmente sendo este seguimento iniciado 2 a 3 meses após o término do tratamento radioterápico; oportunidade na qual se discute abordagem cervical de pacientes inicialmente positivos que apresentem resposta após tratamento irradiante e quimioterápico, além de avaliação do tumor primário.

Considerando o papel estabelecido para avaliação precoce em outros tumores por FDG- PET, Kostakoglu et. al avaliaram prospectivamente 47 pacientes com diagnósticos de linfoma difuso de grandes células B e Linfoma de Hodgkin através da realização de um exame prévio e reavaliação precoce com novo FDG-PET após 1 ciclo de quimioterapia. Dentre os 31 pacientes com resultado negativo ao FDG-PET realizado após o primeiro ciclo, todos apresentaram resposta sustentada a um seguimento de 28 meses. Dentre os 16 pacientes com exame positivo após o primeiro ciclo, 14 apresentaram doença refratária ou recidiva tumoral no período^24^.

**Objetivo**

Avaliar o papel da queda do SUV entre PET-CT dedicado com 18F-FDG previamente ao início do tratamento, após primeiro ciclo de quimioterapia de indução (no D14) e após término do tratamento quimioradioterápico, em pacientes com carcinoma epidermóide de cabeça e pescoço estádios III ou IV não metastáticos à distância ressecáveis ou irressecáveis tratados com quimioterapia neoadjuvante seguido de radioterapia ou quimioradioteapia. Os achados no PET CT serão correlacionados com a taxa de resposta após o 2º ciclo de quimioterapia, após o tratamento quimioradioterápico, controle locoregional, sobrevida livre de progressão e sobrevida global, os quais serão determinados por métodos padrões como exame clínico, endoscópico (se indicado) e por Tomografia Computadorizada.

Deve-se observar que as decisões clínicas serão tomadas de acordo com métodos estabelecidos na política de tratamento vigente, tais quais as observadas por critérios de resposta pela WHO modificada e WHO. Assim, dados relativos ao achados do PET-CT não serão considerados na avaliação de resposta e decisão no prosseguimento do tratamento quimioterápico e radioterápico.

**Casuística**

Trata-se de estudo prospectivo não controlado e unicêntrico que será realizado no Hospital AC Camargo nos Departamentos de Imagem, Oncologia Clínica, Cirurgia de Cabeça e Pescoço e Radioterapia. Serão recrutados 60 pacientes com diagnóstico de carcinoma epidermóide de cabeça e pescoço, estádios III e IV não metastáticos à distância, sem tratamento prévio para a neoplasia.

**Pacientes e Métodos**

Pacientes serão diagnosticados e estadiados por métodos de imagem e propedêutica armada de acordo com prática convencional vigente, conforme descrito abaixo. Até 10 dias antes do início do primeiro ciclo de quimioterapia, no dia 14 do ciclo 1 (tolerável realização de PET-CT entre décimo segundo e décimo sexto dia do ciclo 1 da quimioterapia de indução). Ao término da radioterapia, 8 a 12 semanas após seu término, os pacientes terão opção de realizar terceiro PET-CT se julgado de interesse clínico pelo médico assistente. Observa-se que os investigadores das especialidades oncologia clínica, cirurgia de cabeça e pescoço e radioterapia serão cegos para os resultados dos 2 primeiros PET-CTs realizados, os quais serão avaliado apenas pelo investigador do departamento de imagem. Neste momento, como parte do “screening” pré-estudo, o investigador locado no Departamento de Imagem deverá prestar a informação aos demais pesquisadores quanto à ausência de qualquer suspeita de disseminação metastática à distância para cada paciente avaliado. Se houver suspeita por parte de qualquer investigador quanto a possibilidade de metástase à distância, informações relativas ao primeiro PET-CT, associadas aos demais exames realizados, deverão ser confrontados. Assim, os critérios de avaliação de resposta e decisões terapêuticas até o término da radioterapia não terão influência dos achados do primeiro nem do segundo PET-CT realizado. As decisões clínicas deverão ser baseadas, até este momento, nos métodos utilizados na prática clínica (exame clínico, exames endoscópicos, se indicados, e tomografia computadorizada). Ao terceiro PET-CT, os demais investigadores terão acesso ao mesmo para auxílio na decisão terapêutica da abordagem do tumor primário e discussão para indicação de esvaziamento cervical.

**Critérios de Inclusão**

Os pacientes poderão ser incluídos no estudo se preencherem todos os seguintes critérios de eligibilidade:

1. Diagnóstico histológico de carcinoma epidermóide primitivo de orofaringe, hipofaringe, laringe.
2. Diagnóstico histológico de carcinoma epidermóide primitivo de cavidade oral avançado considerado irressecável.
3. Faixa etária entre 18 e 70 anos.
4. Tumores dos estádios T2(N+) a T4, N0 a N3, M0.
5. Candidatos tratamento cirúrgico radical por apresentarem doença loco-regional ressecável radicalmente.
6. Pacientes com doença locoregional avançada irressecável, sem metástase a distância.

Durante avaliação inicial, cirurgião deverá registrar ressecabilidadde (discriminar se paciente apresenta tumor considerado ressecável ou irressecável).

1. Condições clinicas que permitam tanto cirurgia radical (ASA I a III) como quimioterapia neoadjuvante com compenentes docetaxel, cisplatina e 5-fluorouracil ou radioterapia associada a quimioterapia com platina.
2. Ausência de tratamentos prévios do tumor como cirurgia, radioterapia ou quimioterapia. Biópsia prévia da lesão e traqueostomias serão permitidas.
3. Condição de desempenho (escala de Karnofsky) de 70 ou mais.
4. Expectativa de vida estimada de pelo menos 6 meses.
5. Reserva medular adequada: leucócitos > 3,500 / mm3 , neutrófilos > 1,500/mm3, plaquetas > 100,000/ mm3; hemoglobina > 9,0 g/dl.
6. Função hepática adequada: bilirrubina total < 1.5 vezes o limite superior normal; TGO e TGP < 3 vezes o limite superior normal.
7. Função renal adequada: creatinina < 1.5 vezes o limite superior normal, clearance de creatinina 60ml/1,73m2/24h ou maior .
8. Aderência do paciente e proximidade geográfica que permitam acompanhamento adequado.
9. Aceitação para participar do estudo como voluntãrio e assinatura de consentimento pós-informado.

**Critérios de Exclusão**

1. Doença cardíaca ativa ou não compensada por tratamento, ou infarto agudo do miocárdio nos 6 meses prévios.
2. Infecção ativa presente (ao critério do investigador).
3. Doenças sistêmicas concomitantes consideradas sérias pelo investigador.
4. Outra neoplasia primária (exceto carcinoma in situ da cérvix uterina ou carcinoma basocelular de pele adequadamente tratado), exceto se após tratamento curativo (sem radioterapia ou cirurgia cervico-facial), há mais de 5 anos.
5. Presença de doença psiquiátrica severa.
6. Participação em outro protocolo com droga experimental

**Seleção pré-estudo**

a- Obtenção do Consentimento Informado do Paciente, antes da entrada no estudo.

b- História e exame físico (peso, altura, condição de desempenho (Karnofsky), sinais vitais).

c- Exame hematológico (Hemograma c/ plaquetas)

d- Bioquímica (Na, K, Mg, Uréia, creatinina, Ca, albumina, fosfatase alcalina, TGO, TGP, LDH, bilirrubinas, urinálise, teste de gravidez para mulheres); depuração de creatinina (clearance de creatinina 60ml/1,73m2/24h ou maior).

1. e- Tomografia computadorizada ou ressonancia magnetica cervical. Raio X de tórax para os casos com pescoço estadiado como N0 ou N1 e Tomografia Computadorizada de tórax para os estadiados com N2 ou N3.

Neste momento, exames convencionais utilizados no estadiamento do paciente serão analisados pelos investigadores. O PET-CT inicial será avaliado pelo investigador da imagem, e o mesmo prestará informações relativas a seus achados somente se houver suspeita de metástase à distância. Da mesma forma, qualquer suspeita de metástases a distância por parte de qualquer dos investigadores levarão ao acesso de todos os investigadores ao achado do primeiro PET-CT.

f- Exame loco-regional de cabeça e pescoço.

1. Biópsia confirmando o diagnóstico histopatológico de carcinoma epidermóide de cabeça e pescoço.

**Tratamento**

Os pacientes receberão tratamento de quimioterapia associado a radioterapia no modo neoadjuvante, sendo a quimioterapia de indução constituída por um dos regimes frequentemente utilizados na prática clínica de acordo com evidência disponível para esta modalidade terapêutica, que associa taxanes (paclitaxel ou docetaxel) a cisplatina e 5-fluorouracil infusional, seguindo-se de radioterapia isolada ou associada a quimioterapia baseada em platina, conforme exemplos listados abaixo:

1- Paclitaxel 175mg/m² EV D1, Cisplatina 100mg/m² EV D1 e 5FU 500mg/m² EV infusão contínua D2 a D6 . O esquema quimioterápico será repetido a cada 3 semanas por 3 ciclos^13^.

2- Docetaxel 75 mg/m^2^ EV D1, Cisplatina 75 mg/m^2^ EV D1 e 5FU 750mg/m^2^ EV infusão contínua D1 a D5. Antibioticoterapia profilática: Ciprofloxacina 500mg a cada 12 horas por 10 dias consecutivos, iniciado no D5. O esquema quimioterápico será repetido a cada 3 semanas por até 4 ciclos^15^.

3- Docetaxel 75 mg/m^2^ EV D1, Cisplatina 100 mg/m^2^ EV D1 e 5FU 1000mg/m^2^ EV em infusão contínua D1 a D4. Antibioticoterapia profilática: Ciprofloxacina 500mg a cada 12 horas por 10 dias consecutivos, iniciado no D5. O esquema quimioterápico será repetido a cada 3 semanas por 3 ciclos ^16^.

Fatores estimuladores de crescimento de colônia de granulócitos serão utilizados se neutropenia febril ou se mantiver neutropenia no D28.

**Avaliação de resposta**

Ao segundo ciclo, pacientes serão submetidos a avaliação de resposta por imagem de acordo com critérios da Organização Mundial da Saúde modificado(mWHO) e Organização Mudial da Saúde WHO, conforme anexo abaixo. Pacientes que apresentem pelo menos resposta parcial, com redução de pelo menos 25% no produto dos maiores diâmetros das lesões alvo deverão receber todo o curso de terapia previsto, que compreende total de 3 ou 4 ciclos a depender de protocolos utilizados na prática clínica, seguido de tratamento local com radioterapia com ou sem platina radiosensibilizante.

**Radioterapia**

Será administrado 70 Gy como dose total de radioterapia, 1,8 Gy em fração única diária por 5 dias/semana, durante 7 e 1/2 semanas. O tratamento será efetuado em acelerador linear de 4 MeV em campos englobando a região cervicofacial até 50,4 Gy, com proteção medular aos 45 Gy, e a seguir 2 reduçõ**e**s sobre a lesão primária aos 50,4 e 64 Gy. O “boost” final, após 64 Gy, deverá englobar a lesão primária com margens de 1 cm.

Para administração da radioterapia o paciente será imobilizado com máscara na mantido em posição supina. Nódulos cervicais, quando presentes devem ser identificados com chumbo. Serão utilizados campos anteriores para o tratamento da região cervical inferior e fossas supraclaviculares e campos laterais opostos para tratamento do tumor primário.

Os pacientes receberão, à critério do oncologista clínico, uma das seguintes opções:

1– Radioterapia isolada

2- Radioterapia associada à Carboplatina AUC 1,5 [Dose em mg= CLcr calculado + 25)x1,5] semanalmente, por no máximo 7 semanas.

3- Radioterapia associada à Cisplatina 100mg/m² D1, D22 e D43.

**Cirurgia**

Todos os pacientes arrolados no estudo deverão ser submetidos a uma avaliação cirúrgica prévia ao início do tratamento, que deve constar de:

1. Exame loco-regional de cabeça e pescoço, incluindo exame da cavidade oral, orofaringe, laringoscopia indireta ou laringofibroscopia e palpação cerivical.
2. Tomografia computadorizada ou ressonância magnética de faringe e pescoço sem e com contraste.

Esta avaliação deve ocorrer num período não superior a 4 semanas do início do tratamento.

Os pacientes deverão ser reavaliados com exame clínico, nasofaringoscopia e tomografia ou ressonância após, no máximo de 2 semanas após a última aplicação de 2 ciclos de quimioterapia neoadjuvante, na quinta semana de tratamento e entre 6 a 12 semanas após o término da radioterapia. Os pacientes com resposta observada inferior a uma resposta parcial (doença estável ou progressão do tumor) nas avaliações durante a quimioterapia de indução e na quinta semana da radioterapia, serão avaliados para ressecção cirúrgica do tumor.

Do mesmo modo, nos pacientes com resposta parcial ou completa, esvaziamento radical ou radical modificado será efetuado em todos os casos de N2 (a,b ou c) ou N3 ao diagnóstico. Esvaziamento supraomohióideo ampliado para nivel IV será realizado nos casos N0 e N1 submetidos a cirurgia de resgate após insucesso da quimioterapia neoadjuvante ou da raditorerapia aos 5040 cGy.

A técnica cirúrgica empregada consiste basicamente de:

1. Retalho cervical incluindo o platisma. Extensão para fossa supraclavicular em casos de esvaziamentos radicais.
2. Esvaziamento cervical contralateral quando indicado.
3. Esvaziamento cervical homolateral quando indicado.
4. Ressecção do tumor primário quando indicado (resgate).
5. Reconstrução com retalho miocutâneo ou transplante microcirúrgico, quando se ressecar mais de 50% da mucosa da faringe.

**Avaliação ao término do tratamento**

1. Realização de avaliação por imagem com Tomografia computadorizada ou Ressonância Nuclear Magnética de pescoço 6 semanas ao término da radioterapia. Na reavaliação pelo investigador /médico assistente do Departamento de Cirurgia de Cabeça e Pescoço serão discutidos aspectos relativos a abordagem cirúrgica do pescoço e tumor primário.

2. Pacientes cujo médico assistente entender haver potencial benefício para uso de PET-CT 8 a 12 semanas após termino de radioterapia terão opção de proceder exame, que será avaliado pelo cirurgião de cabeça e pescoço e oncologista clínico, em momento de início de seguimento pós-radioterapia.

**Seguimento dos pacientes no período pós-tratamento**

a) Mensal nos primeiros 6 meses;

b) Bimestral por 6 meses adicionais;

c) Trimestral por 2 anos adicionais;

d) Semestral por 2 anos adicionais.

Exames de imagem anuais (tomografia computadorizada ou ressonância magnética cervical e RX de tórax) ou conforme indicado.

**Descontinuações**

Os motivos da descontinuação deverão ser documentados.

**Anatomia-patológica**

**Pré-tratamento :**

1. Material de biópsia corado em HE com gradação histológica.
2. Matarial de biópsia mantido em banco de tumores

**Pós-tratamento**

1. Biópsias e material de cirurgias de resgate corados pelo HE

**Exame das peças operatórias**

1. Todas as peças de ressecção do tumor primário serão estudadas em cortes seriados
2. Todos os linfonodos dissecados serão classificados segundo o nível, número, diâmetro e presença de ruptura de cápsula.
3. No tumor primário será estudado histologicamente, descrevendo-se o grau de diferenciação, presença de necrose, infiltração perineural e embolização vascular.

**Medidas de eficácia**

**Mensurabilidade de doença**

**Lesões bidimensionalmente mensuráveis**

a- Exame físico: nódulo ou linfonodo superficial com pelo menos 20X10 mm.

1. Tomografia computadorizada: lesão de pelo menos 10 X 10 mm

**Lesões unidimensionalmente mensuráveis**

Incluem todas as lesões em que se pode medir apenas um diâmetro

a- Exame físico: > 20 mm

b- Tomografia computadorizada : > 20 mm

**Lesões avaliáveis não mensuráveis**

Lesões não uni ou bidimensionalmente mensuráveis como definido acima (e.x.: tumorações primárias).

**Critérios para avaliação de resposta**

**Quanto à resposta**

Todos os pacientes que receberem > 2 ciclos de quimioterapia neoadjuvante e naqueles com resposta, uma segunda avaliação será feira na quinta semana de radioterapia.

**Critérios de resposta - WHO e WHO modificada**

Todas as lesões uni ou bidimensionalmente mensuráveis por exame físico devem ser medidas após o segundo ciclo de quimioterapia, conforme acima mencionado.

Em caso de suspeita de progressão antes do término do tratamento neoadjuvante ou numa segunda etapa durante o tratamento com radioterapia (até 5040 cGy), deve se repetir a oroscopia, nasofaringoscopia e/ou tomografia computadorizada ou ressonância magnética conforme clinicamente indicado. No entanto, para se definir a resposta global do paciente, a mensuração considerada será a efetuada após o término do tratamento.

**Doença uni ou bidimensionalmente mensurável**

**Resposta completa (RC):** desaparecimento de toda doença conhecida, seja clinicamente, radiologicamente ou por endoscopia / biópsias.

**Resposta parcial (RP):** em caso de doença bidimensionalmente mensurável, redução em pelo menos 25% ou 50%, da soma dos produtos dos diâmetros perpendiculares maiores em todas as lesões mensuráveis, de acordo com os critérios WHO modificado e WHO respectivamente. Pacientes que alcançarem resposta parcial sob qualquer dos critérios serão considerados responsivos e seguirão terapia irradiante proposta. Em caso de lesões unidimensionalmente mensuráveis, redução em pelo menos 30% da soma dos diâmetros de todas as lesões. Não é necessário que todas as lesões regridam para qualificar RP, porém nenhuma lesão pode ter progredido ou aparecido.

**Doença estável (DE):** para doença bidimensionalmente mensurável < 50% de diminuição e < 25% de aumento no tamanho da soma dos produtos dos diâmetros perpendiculares maiores de todas as lesões mensuráveis observadas antes do tratamento. Para doença unidimensionalmente mensurável, < 50% de redução e < 25% de aumento da soma do diâmetro de todas as lesões observadas antes do tratamento. Não devem aparecer novas lesões.

**Doença progressiva (DP):** > 25% de aumento de tamanho de pelo menos uma lesão mensurável em comparação às medidas basais.

**Doença não mensurável**

**Resposta completa:** desaparecimento completo de toda a dça conhecida.

**Resposta parcial:** redução estimada do tamanho do tumor de 50%.

**Doença estável:** diminuição estimada de menos de 50% ou aumento estimado menor que 25%.

**Doença progressiva:** aparecimento de quaisquer lesões novas não identificadas previamente ou aumento estimado maior que 25% em lesões existentes.

**Resposta patológica**

**Resposta completa:** resposta completa documentada em biópsias ou peça cirúrgica.

**Resposta parcial:** presença de doença na peça cirúrgica ou em biópsias, em pacientes com resposta completa ou parcial constatadas antes da cirurgia.

**Determinação da resposta global**

| **Resposta em lesões bidimensionalmente mensuráveis** | **Resposta em lesões unidimensionalmente mensuráveis** | **Resposta em lesões não mensuráveis** | **Resposta global** |
| --- | --- | --- | --- |
| DP | qualquer | Qualquer | DP |
| qualquer | DP | Qualquer | DP |
| DE | qualquer exceto DP | Idem | DE |
| RP | qualquer exceto DP | Idem | RP |
| RC | RC | RC | RC |
| RC | qualquer exceto DP e RC | Idem | RP |
| Qualquer | qualquer | DP ou lesão nova | DP |
| Ausente | RP | qualquer exceto DP | RP |
| Ausente | RC | Qualquer exceto DP e RC | RP |
| Ausente | RC | RC | RC |

**Duração da resposta**

A duração da melhor resposta será desde a data do início do tratamento até documentação da progressão de doença.

**Tempo para progressão de doença**

Desde a data do início do tratamento até progressão, óbito, último contato ou última avaliação do tumor antes do início de tratamento antitumoral posterior (resgate).

**Eventos adversos**

Qualquer evento indesejável associado à utilização de um medicamento, sendo ou não considerado relacionado ao medicamento, e inclui qualquer efeito colateral, toxicidade ou reações de sensibilidade. Inclui qualquer evento clínico ou laboratorial não normalmente observado no paciente.

Serão classificados e graduados com os critérios expandidos de toxicidade comum do NCI (Apêndice).

Correções de dose do quimioterápicos bem como descontinuação de pacientes do estudo ficarão a critério dos investigadores. A tomada de qualquer das atitudes acima deverá ser documentada no prontuário e ficha clínica do paciente.

**Definições**:

**Taxa de preservação (a ser avaliada em pacientes considerados candidatos a cirurgia com intuito curativo ao diagnóstico)**: obtenção de controle local doença sem necessidade de intervenção cirúrgica sobre o sítio primário da doença. Será avaliada 4 semanas após o término da radioterapia (7040 Gy).

**Toxicidade proibitiva**: evento letal ou evento adverso grave, que resulte em necessidade de hospitalização para tratamento.

**Análise estatística**

Razões simples serão utilizadas para descrever as distribuições de variáveis categóricas. As distribuições de variáveis numéricas serão descritas utilizando-se as medianas e os intervalos (*range*). Curvas de sobrevida serão geradas a partir do método de Kaplan-Meier e comparadas entre diferentes grupos através do método de log-rank. Método de Bootstrap será utilizado para realização de validação interna dos achados. O método de estatística máxima padronizada do log-rank será utilizado para avaliação do *cut-off* ideal para queda do SUV para se considerar um paciente como responsivo. Modelos proporcionais de risco de Cox univariados serão gerados para o cálculo dos Hazard Ratios (e respectivos intervalos de confiança). A análise estatística será realizada nos Programas SPSS e Project R.

**Referências**

1. Jemal A, Siegel R, Ward E, et al. Cancer statistics. CA Cancer J Clin 2007;57:43-66.

2. Seiwert TY, Cohen EE. State-of-the-art management of locally advanced head and neck cancer. Br J Cancer 2005;92:1341-8.

3. Kowalski LP, Franco EL, Torloni H, Fava AS, et al. Lateness of diagnosis of oral and oropharyngeal carcinoma: factors related to the tumour, the patient and health professionals. Eur J Cancer B Oral Oncol. 1994;30B:167-73.

4.Carole Fakhry et al.Clinical Implications of Human Papillomavirus in Head and Neck Cancers. J Clin Oncol 2006; 24: 2606.

5. Jeremic B, et al: Hyperfractionated radiation therapy with or without concurrent low-dose daily cisplatin in locally advanced squamous cell carcinoma of head and neck: A prospective randomized trial. J Clin Oncol 2000; 18(7): 1458-1464.

6. Wendt TG, et al: Simultaneous Radiochemotherapy versus Radiotherapy Alone in Advanced Head and Neck Cancer: A Randomized Multicenter Study. J Clin Oncol 1998, 16: 1318-1324.

7. Pignon JP, et al: Chemotherapy added to locoregional treatment for head and neck squamous-cell carcinoma: Three meta-analyses of updated individual data. Lancet 2000; 355 (9208): 949-955.

8. The Department of Veterans Affairs Laryngeal Cancer Study Group. Induction chemotherapy plus radiation compared with surgery plus radiation in patients with advanced laryngeal cancer. N Engl J Med 1991; 324:1685-90

9. Forastiere AA, et al: Concurrent Chemotherapy and Radiotherapy for Organ Preservation in Advanced Laryngeal Cancer. N Engl J Med 349 (22): 2091-8, 2003.

10. Lefebvre JL, et al: Larynx preservation in pyriform sinus cancer: Preliminary results of a European Organization for Research and Treatment of Cancer phase III Trial. EORTC Head and Neck Cancer Cooperative Group. Natl Cancer Inst 1996;88 (13):890-899.

11. Domenge C, et al: Randomized trial of neoadjuvant chemotherapy in oropharyngeal carcinoma. French Groupe d’Estude des Tumeurs de la Tete et du Cou (GETTEC). Br J Cancer. 83 (12) : 1594-1598, 2000.

12. Calais G, et al: Final Results of the 94–01 French Head and Neck Oncology and Radiotherapy Group Randomized Trial Comparing Radiotherapy Alone With Concomitant Radiochemotherapy in Advanced-Stage Oropharynx Carcinoma. J Clin Oncol 2004; 22(1):69-76.

13. Hitt R, López-Pousa A, Martínez-Trufero J, et al. Phase III Study Comparing Cisplatin Plus Fluorouracil to Paclitaxel, Cisplatin, and Fluorouracil Induction Chemotherapy Followed by Chemoradiotherapy in Locally Advanced Head and Neck Cancer. J Clin Oncol 2005; 23:8636-8645.

14. Schrijvers D, et al: Docetaxel, cisplatin and 5-fluorouracil in patients with locally advanced unresectable head and neck cancer: a phase I-II feasibility study. Ann Oncol 2004; 15:638-645.

15. Vermorken JB, Remenar E, van Herpen C, et al. Cisplatin, Fluorouracil, and Docetaxel in Unresectable Head and Neck Cancer. N Engl J Med 2007; 357:1695-704.

16. Posner MR, Hershock DM, Blajman CR, et al. Cisplatin and Fluorouracil Alone or with Docetaxel in Head and Neck Cancer. N Engl J Med 2007; 357:1705-15.

17. Hitt R, Grau JJ, Lopez-Pausa A, et al. Randomized phase II/III clinical trial of induction chemotherapy (ICT) with either cisplatin/5-fluorouracil (PF) or docetaxel/cisplatin/5-fluorouracil (TPF) followed by chemoradiotherapy (CRT) vs. crt alone for patients (pts) with unresectable locally advanced head and neck cancer. Journal of Clinical Oncology, 2006 ASCO Annual Meeting Proceedings Part I. Vol 24, No. 18S (June 20 Supplement), 2006: 5515.

18. Lorch JH, Posner MR, Wirth LJ, et al. Induction Chemotherapy in Locally Advanced Head and Neck Cancer: A New Standard of Care? Hematol Oncol Clin N Am 2008; 22:1155-1163.

19. Therasse P, Arbuck SG, Eisenhauer EA, et al. New Guidelines to Evaluate the Response to Treatment in Solid Tumors. JNCI 2000; 92(3): 205-16.

20. Miller AB, Hoogstraten B, Staquet M, et al. Reporting results of cancer treatment. Cancer 1981; 47(1):207-14.

21. Burton C, Ell P, Linch D. The role of PET imaging in lymphoma. Br J Haematol. 2004 Sep;126(6):772-84. Review.

22. Beyer T, Townsend DW, Blodgett TM. Dual-modality PET/CT tomography for clinical oncology. Q J Nucl Med. 2002 Mar;46(1):24-34.

23. von Schulthess GK, Steinert HC, Hany TF. Integrated PET/CT: current applications and future directions. Radiology. 2006 Feb;238(2):405-22.

24. Kostakoglu L, Goldsmith SJ, Leonard JP et al. FDG-PET after 1 cycle of therapy predicts outcome in diffuse large cell lymphoma and classic Hodgkin disease. Cancer. 2006 Dec 1;107(11):2678-87.

**Termo de Consentimento Livre e Esclarecido (TCLE)**

**Hospital A.C.Camargo- Fundação Antônio Prudente**

**Rua Professor Antonio Prudente, 211 Telefone : (011) 2189-5000**

**SãoPaulo-SP CEP:01509-010**   

**CONSENTIMENTO PÓS-INFORMADO**

(Obrigatório para Pesquisa Clínica em Seres Humanos- Resolução no 196/96 e resolução CNS 252/97 do Ministério da Saúde)

Este termo está sendo solicitado exclusivamente para participação nesta pesquisa, sem possibilidade de extensão da mesma autorização para outros projetos.

PROJETO: **USO DO PET-SCAN COMO PREDITOR DE EFICÁCIA PARA CONTROLE LOCOREGIONAL EM CARCINOMA EPIDERMÓIDE DE CABEÇA E PESCOÇO.**

1. Dados de identificação do paciente ou responsável legal

**Nome do(a) paciente : ____________________________________________**

**Sexo : masculino ( ) feminino ( ) Data de nascimento _/_/_**

**Documento de identidade no: __________________________**

**Endereço:_______________________________________________**

**Cidade:_________________________Estado:___________**

**CEP:_________________ Tel.:________________________**

**Responsável: ______________________________________________**

**Sexo : masculino ( ) feminino ( ) Data de nascimento _/_/_**

**Documento de identidade no: __________________________**

**Endereço:_________________________________________________**

**Cidade:_________________________Estado:___________**

**CEP:_________________ Tel.:________________________**

1. Objetivos do estudo

Você foi convidado a participar deste estudo em câncer de cabeça e pescoço. O tratamento do câncer de cabeça e pescoço localmente avançado é baseado em quimioterapia com cisplatina e 5-fluoro-uracil concomitante a taxanes seguido de radioterapia concomitante ou não a quimioterapia. O Corpo Clínico do Departamento de Oncologia Clínica do Hospital AC Camargo está desenvolvendo pesquisa clínica na área de câncer de cabeça e pescoço localmente avançado submetidos a tratamento padrão de quimioterapia (cisplatina e 5-fluro-uracil associado a taxane) seguido de radioterapia concomitante ou não a quimioterapia. O objetivo é identificar fatores de resposta precoce ao tratamento e futuramente correlacionar com sobrevida livre de recaída com exames de imagem funcional (PET-CT) e comparar com a tomografia computadorizada, exame de imagem tradicional para avaliar resposta ao tratamento.

3. Descrição do procedimento e duração da participação no estudo

Constitui rotina no Departamento de Oncologia Clínica o esquema de tratamento proposto neste estudo, assim como os exames laboratoriais e de imagem convencionais propostos, não sendo considerado nenhum experimento. Será solicitado adicionalmente realização de exame PET-CT, que é um exame de imagem que associa imagens de metabolismo da glicose com imagens anatômicas da tomografia computadorizada sem contraste, pré terapia, após o 1º ciclo de quimioterapia e após o término da radioterapia. Serão 3 ou 4 ciclos de quimioterapia a cada 21 dias cada. Você sairá do estudo caso apresente progressão da doença ou toxicidade que impeça a continuação da terapia. O PET-CT é expiremental nesse estudo, porém não é expiremental na oncologia, já fazendo parte da propedêutica diagnóstica oncológica, realizado no Departamento de Imagem/ Medicina Nuclear da mesma instituição e com risco baixo de complicações inerentes ao exame. No dia da realização do exame, em jejum, você receberá a administração venosa de um material específico e encomendado previamente que é denominado 18F-FDG e produzido pelo IPEN-CNEN-SP. Este material é utilizado regularmente no setor de Medicina Nuclear para a realização deste exame sem quaisquer complicações e será doado pelo IPEN para a realização do exame. Antes da injeção venosa do 18F-FDG você terá a glicemia verificada através da realização de um exame denominado hemoglicoteste que utiliza punção capilar digital para certificar-se de que a glicemia encontra-se em níveis menores do que 200 mg/dl, pois acima destes valores a qualidade do exame pode estar prejudicada.

Você deverá dispor de pelo menos 3 horas do seu dia para a realização do exame previamente marcado, pois deverá permanecer cerca de 30 minutos em repouso antes da injeção do 18F-FDG, aguardar cerca de 90 minutos para iniciar o exame após a injeção do 18F-FDG e cerca de 50 minutos a 1 hora para a obtenção das imagens do exame e verificação da sua qualidade técnica. Ressalto, porém, que este período poderá ser estendido se houver atraso no horário de entrega do material à instituição e há orientação rotineira no agendamento deste exame no setor aos pacientes que realizam este tipo de exame para que não assumam compromissos com horários previstos neste dia.

Riscos potenciais

Riscos inerentes do tratamento habitual, mesmo não participando desse protocolo:

Efeitos colaterais mais freqüentes pelo uso de quimioterapia:

- Docetaxel: náuseas, vômitos, mucosite, queda de glóbulos brancos e infecção, dor muscular e queda de cabelo.

- 5 fluoraucil: mucosite, diarréia, síndrome palmo-plantar, pancitopenia, isquemia miocárdica, queda de cabelo grau leve

- Cisplatina: náusea, vômito, toxicidade renal, toxicidade auditiva, neuropatia, isquemia, queda de glóbulos vermelhos, queda de glóbulos brancos ou de plaquetas.

- Carboplatina: náusea, vômito, toxicidade renal, toxicidade auditiva, queda de glóbulos vermelhos, queda de glóbulos brancos ou de plaquetas.

Efeitos colaterais relacionados com a Radioterapia:

- mucosite, infecção odontogênica, dermatite ou necrose óssea.

Efeitos relacionados a tomografia computadorizada:

- reação alérgica ao contraste, flebite.

Quanto ao PET-CT, que será o único procedimento adicional ao padrão de terapia e exames que o paciente normalmente receberia fora do estudo, os riscos previstos são aqueles envolvidos com a sensação de dor que poderá ocorrer quando for realizada a punção capilar digital com agulha de insulina para a verificação da glicemia e na punção venosa com escalpe para a injeção do 18F-FDG. Há, ainda, o risco de extravasamento dérmico do material injetado venosamente mas , que caso ocorra, não implicará em nenhuma lesão de órgão e, também, o risco de possível infecção após as punções citadas, mas que são minimizadas com assepsia adequada.

Pacientes do sexo feminino, em idade fértil, devem assegurar que não engravidarão durante a terapia devido ao potencial teratogênico das drogas e da radiação (exames de imagem), portanto assumindo os riscos da gestação , caso venha a acontecer.

Benefícios previstos

A participação neste estudo tem como propósito melhorar a abordagem de tratamento dos pacientes com câncer de cabeça e pescoço localmente avançados. Caso você concorde em participar do estudo pode haver ou não benefício direto para você. Esperamos que as informações obtidas neste estudo com a inclusão do método de imagem (PET-CT) precocemente na avaliação destes pacientes permita identificar pacientes que realmente se beneficiarão do tratamento quimioterápico seguido por radioterapia e, em estudos futuros, isto possa ser confirmado e evitar toxicidade adicional desta drogas em pacientes que não obtenham a resposta adequada esperada com o tratamento inicial instituído.

Tratamentos alternativos ao objeto da pesquisa

Quando optado pelo tratamento quimioterápico seguido de radioterapia associado ou não a quimioterapia, os esquemas e doses serão os tradicionais utilizados na nossa instituição. O tratamento oferecido não está em teste.

Salvaguarda de confidencialidade, sigilo e privacidade

A eventual inclusão dos resultados em publicação científica será feita de modo a manter seu anonimato. Você terá acesso aos seus dados de exames, atendimentos médicos e administração de terapia, quando solicitados.

Esclarecimentos sobre compensações ou danos relacionados à pesquisa

Você não terá nenhum tipo de remuneração ao aceitar participar deste estudo. A pesquisa não envolve nenhuma forma de compensação financeira aos participantes. Não existe nenhum tipo de indenização para complicações causadas pelo tratamento.

Esclarecimentos sobre outros direitos do paciente sujeito à pesquisa

A sua participação no estudo é voluntária. Você tem o direito de sair do estudo a qualquer momento e por qualquer motivo. Caso venha a abandonar o estudo ou decidir não participar do mesmo, o seu tratamento não será prejudicado. No entanto, se você decidir sair da pesquisa, deverá informar ao seu médico.

O Comitê de Ética Médica do Hospital A.C.Camargo é o responsável legal para a certificação de que os direitos dos pacientes estejam protegidos. Este comitê analisou e aprovou este estudo.

Informações sobre nomes, telefones e endereços para contatos

Esclarecimentos para questões sobre os direitos dos participantes na pesquisa e/ou danos relacionados à pesquisa, contatar os pesquisadores Dr Ulisses Ribaldo Nicolau (011) 99011147 ou 21895000 r 1551 ou Dr Tadeu Ferreira de Paiva Júnior (011) 84913815 ou 21895000 r 1551. Se o pesquisador principal não fornecer as informações/ esclarecimentos suficientes, por favor, entre em contato com o coordenador do Comitê de Ética do Hospital A.C.Camargo-SP, pelo telefone: 2189-5020.

Você receberá cópia deste documento e o original será arquivado no prontuário do médico. Somente assine este documento se consentir integralmente com seus termos.

**Consentimento Livre e Pós-informado**

Eu declaro que li e compreendi o procedimento. Declaro também que discuti este procedimento com  meu médico. Eu entendo o propósito do estudo e os métodos que serão utilizados. Entendo também que a minha entrada neste estudo é voluntária.

Assinatura do (a) paciente ou responsável legal ___________________________

Data: ____/ ____ /____

Nome do(a) paciente ___________________________________ RG ___________

**DECLARAÇÃO DO MÉDICO OBTENDO CONSENTIMENTO PÓS-INFORMADO**

Eu declaro que expliquei este procedimento, com todos os detalhes necessários para o(a) paciente (ou seu responsável legal) ___________________________________.

No meu julgamento, houve acesso a todas as informações disponíveis, incluindo os riscos e benefícios para que se possa fazer uma decisão informada.

Assinatura do Médico __________________________  Data: _____ / ____ / ____
